# Supplementary figures and images for: Soil organic carbon fraction accumulation and bacterial characteristics in curtilage soil: Effects of land conversion and land use
Source: PLoS One. 2023 Apr 6;18(4):e0283802. doi: 10.1371/journal.pone.0283802 (PMC10079021; doi:10.1371/journal.pone.0283802)

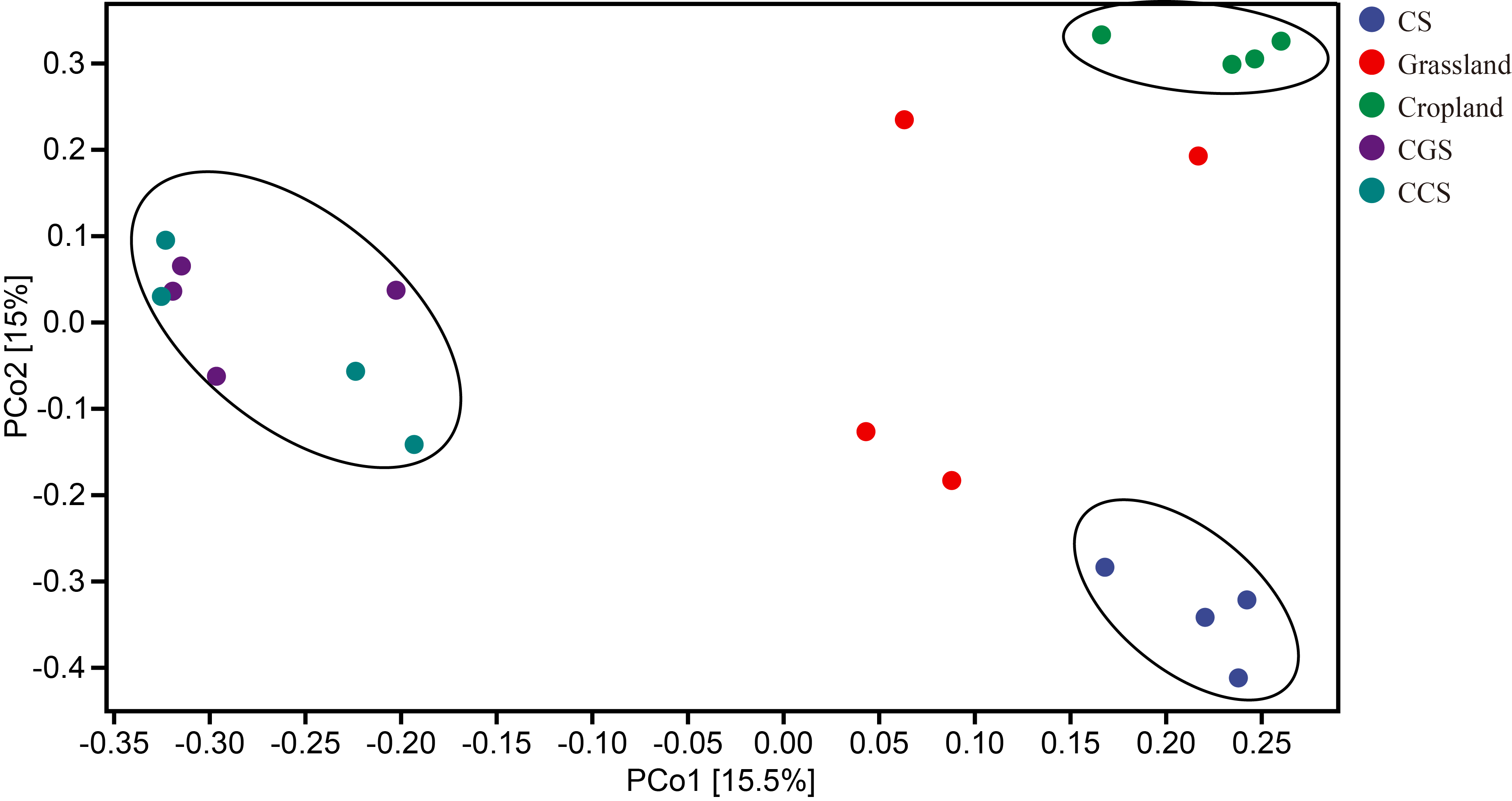

Supplement: S1 Fig — (TIF) [file pone.0283802.s001.tif]

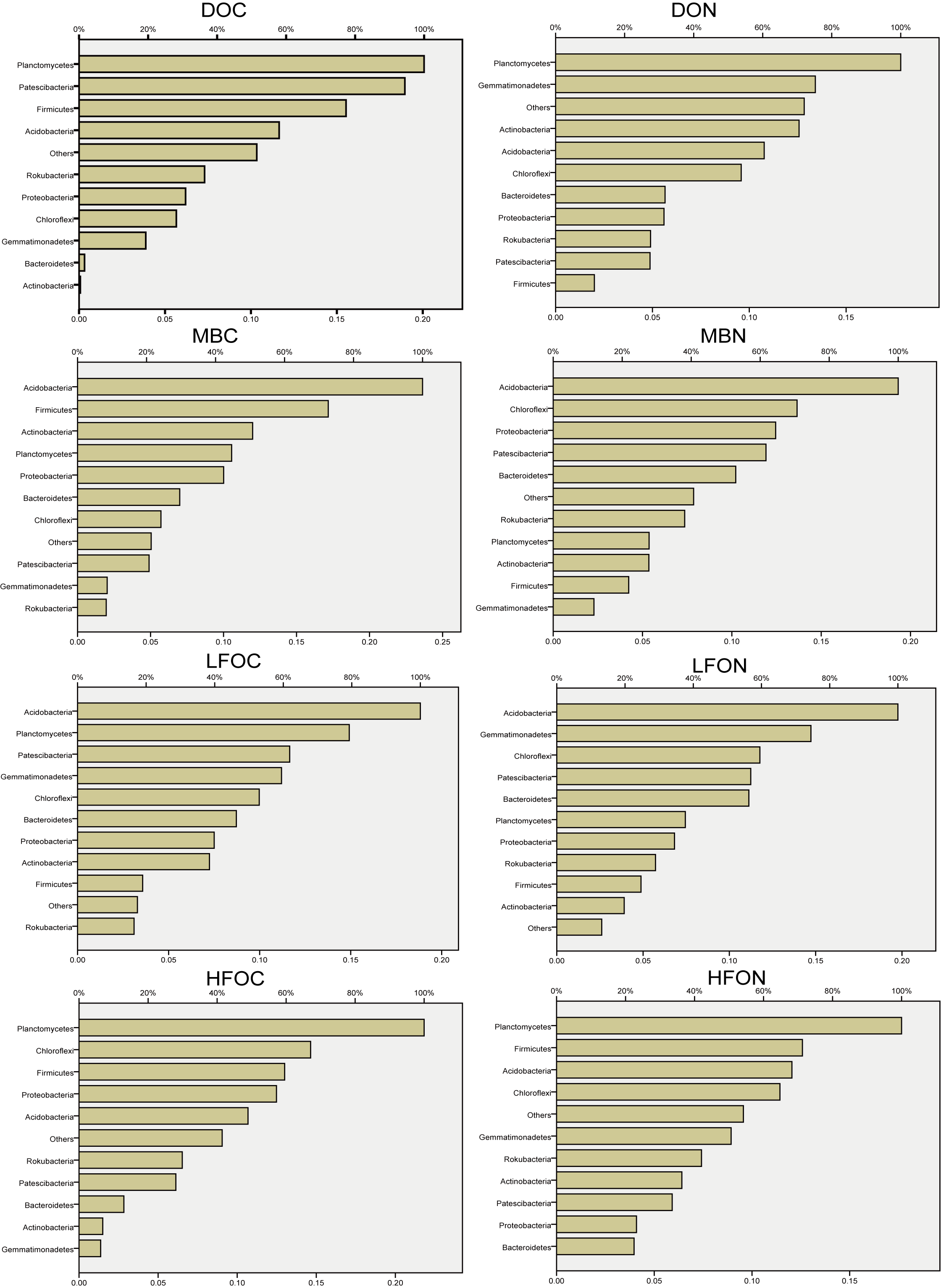

Supplement: S2 Fig — (TIF) [file pone.0283802.s002.tif]

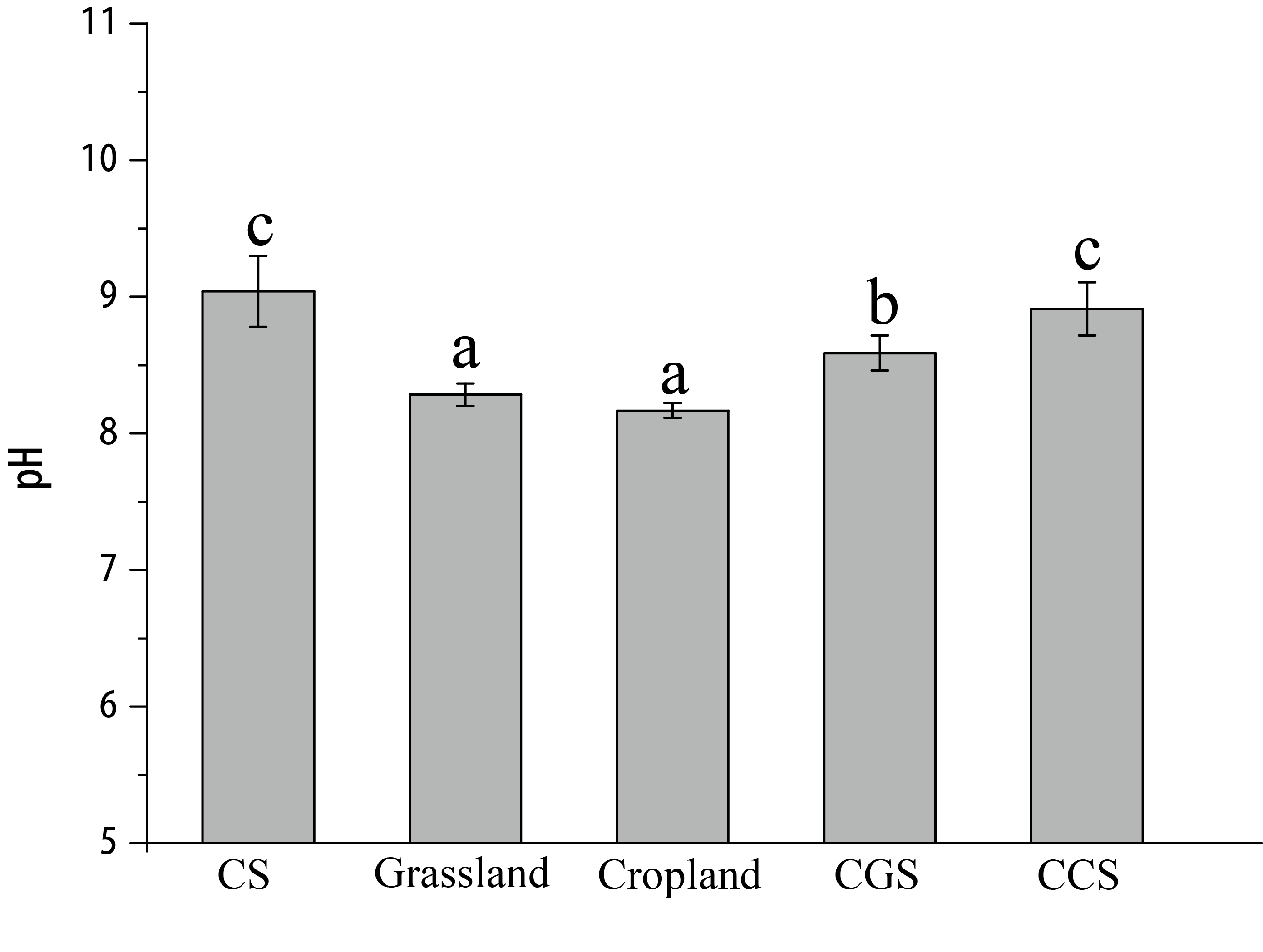

Supplement: S3 Fig — Different letters on bars indicated significant differences among the soil types analyzed by Duncan test. (TIF) [file pone.0283802.s003.tif]

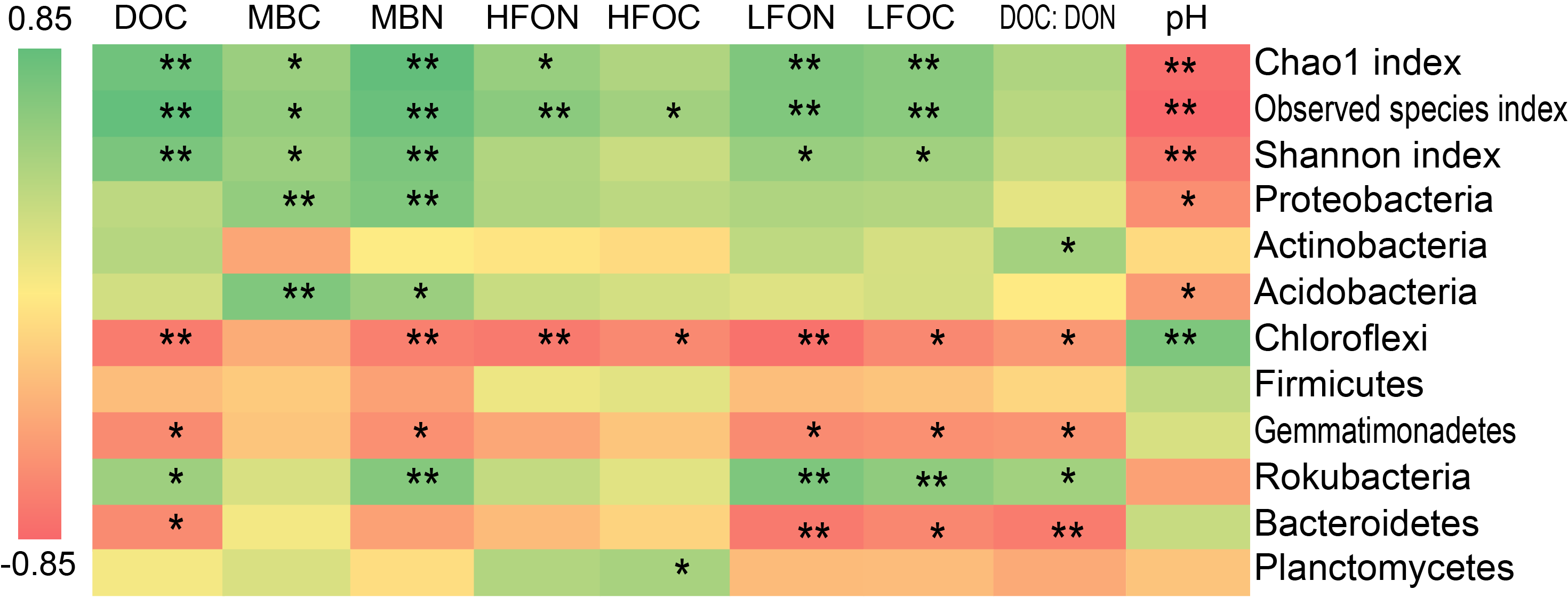

Supplement: S4 Fig — (TIF) [file pone.0283802.s004.tif]

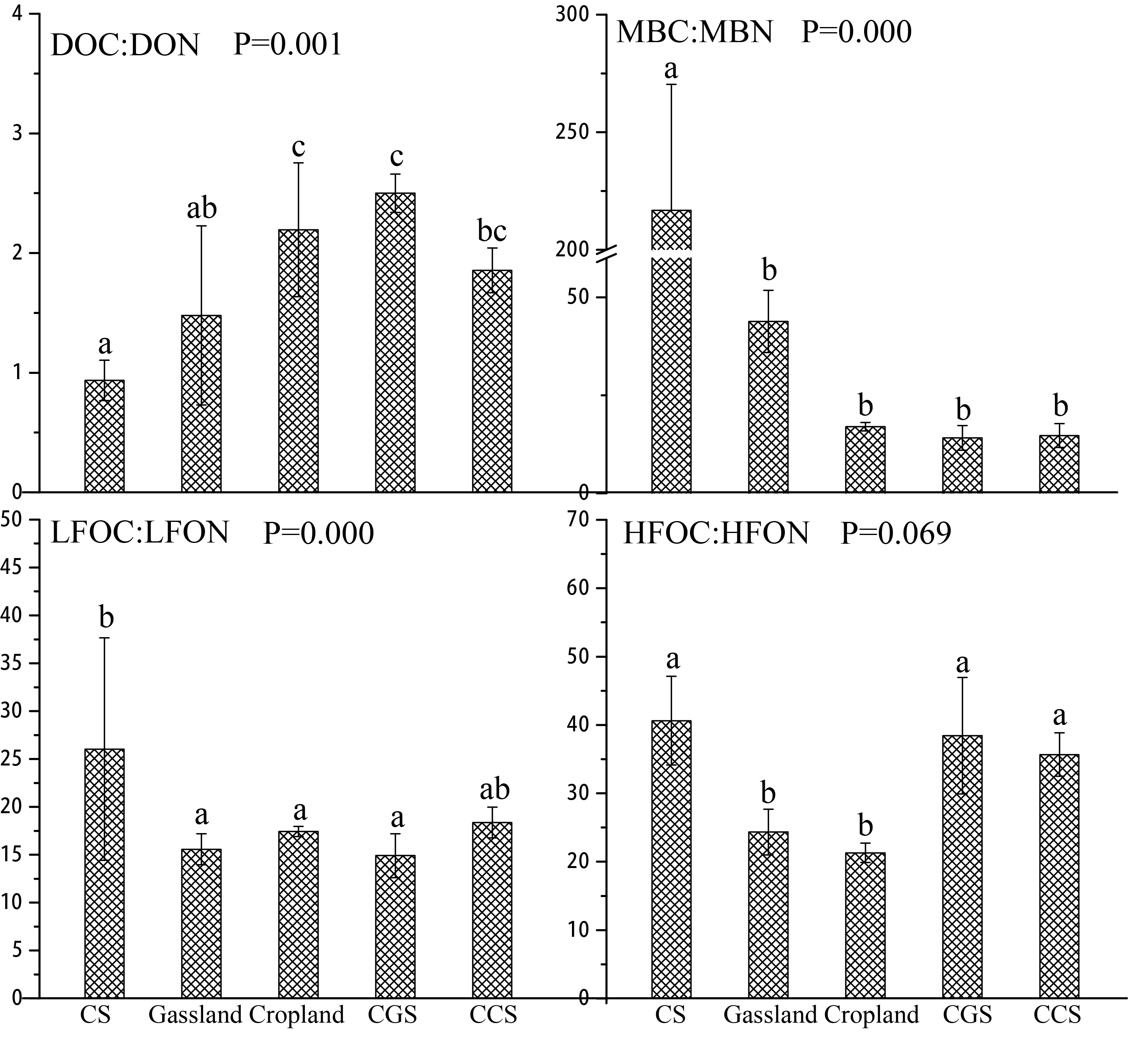

Supplement: S5 Fig — Different letters on bars indicated significant differences among the soil types analyzed by Duncan test. (TIF) [file pone.0283802.s005.tif]
