# Supplementary material for: Soil organic carbon fraction accumulation and bacterial characteristics in curtilage soil: Effects of land conversion and land use
Source: PLoS One. 2023 Apr 6;18(4):e0283802. doi: 10.1371/journal.pone.0283802 (PMC10079021; doi:10.1371/journal.pone.0283802)
Supplement: S1 Table — (DOC) [file pone.0283802.s006.doc]

**S1 Table. The relative abundance of dominant functional genes in the five soil types.**

| Functions | CS | Grassland | Cropland | CGS | CCS |
| --- | --- | --- | --- | --- | --- |
| Amino Acid Biosynthesis | 40463.67 | 39173.95 | 38738.49 | 38012.85 | 38502.87 |
| Aromatic Compound Biosynthesis | 3039.99 | 2739.41 | 2726.76 | 2649.54 | 2728.36 |
| Carbohydrate Biosynthesis | 14513.58 | 13661.15 | 14219.49 | 13388.77 | 13644.7 |
| Cell Structure Biosynthesis | 9291.68 | 8503.67 | 8420.8 | 7995.13 | 8152.51 |
| Cofactor, Prosthetic Group, Electron Carrier, and Vitamin Biosynthesis | 34893.13 | 35787.05 | 35423.95 | 36530.6 | 35664.29 |
| Fatty Acid and Lipid Biosynthesis | 20392.8 | 19952.59 | 22253.22 | 20079.39 | 19946.9 |
| Nucleoside and Nucleotide Biosynthesis | 37652.32 | 35221.55 | 34746.82 | 34163.23 | 34707.05 |
| Secondary Metabolite Biosynthesis | 5979.94 | 5886.56 | 5654.52 | 5578.19 | 5697.58 |
| Amino Acid Degradation | 1748.54 | 2403.9 | 2973.54 | 2937.75 | 2873.95 |
| Aromatic Compound Degradation | 1588.59 | 2699.95 | 3048.47 | 3509.94 | 3323.92 |
| C1 Compound Utilization and Assimilation | 5751.98 | 4959.54 | 4703.53 | 4762.86 | 4771.51 |
| Carbohydrate Degradation | 3553.3 | 4554.25 | 4681.67 | 5084 | 4617.94 |
| Carboxylate Degradation | 2329.15 | 3111.37 | 3250.86 | 3522.3 | 3143.13 |
| Fatty Acid and Lipid Degradation | 1255.87 | 1440.54 | 1587.5 | 1531 | 1620.03 |
| Inorganic Nutrient Metabolism | 2740.39 | 3289.06 | 3219.93 | 3305.36 | 3163.3 |
| Nucleoside and Nucleotide Degradation | 6553.11 | 7035.79 | 7244.74 | 7286.69 | 6982.48 |
| Polymeric Compound Degradation | 1591.28 | 1796.41 | 1875.47 | 1905.69 | 1807.96 |
| Secondary Metabolite Degradation | 1713.24 | 2457.22 | 2748.13 | 2798.72 | 2411.79 |
| Electron Transfer | 4353.7 | 4135.61 | 4138.18 | 3995.4 | 4166.99 |
| Fermentation | 9189.64 | 9908.6 | 9454.37 | 9962.43 | 9341.3 |
| Glycolysis | 4146.06 | 3921.39 | 3782.3 | 3904.69 | 3874.55 |
| Pentose Phosphate Pathways | 3119.38 | 3019.3 | 2989.64 | 2961.1 | 3026.61 |
| Photosynthesis | 2012.55 | 1879.87 | 1916.3 | 1894.86 | 1852.75 |
| Respiration | 4433.19 | 4193.24 | 4241.47 | 4093.42 | 4244.05 |
| TCA cycle | 8024.44 | 8566.97 | 8534.29 | 8466.57 | 8439.4 |
