# Supplementary material for: Soil organic carbon fraction accumulation and bacterial characteristics in curtilage soil: Effects of land conversion and land use
Source: PLoS One. 2023 Apr 6;18(4):e0283802. doi: 10.1371/journal.pone.0283802 (PMC10079021; doi:10.1371/journal.pone.0283802)
Supplement: S2 Table — (DOC) [file pone.0283802.s007.doc]

**S2 Table.** **The Pearson Correlation Analysis among organic matter fractions and pH.**

| Parameters | DOC | DON | MBC | MBN | HFON | HFOC | LFON | LFOC | pH |
| --- | --- | --- | --- | --- | --- | --- | --- | --- | --- |
| DOC | 1 | 0.417 | .474* | .498* | .724** | .681** | .614** | .481* | -.764** |
| DON | 0.417 | 1 | .457* | -0.06 | 0.435 | .519* | -0.102 | -0.126 | -0.263 |
| MBC | .474* | .457* | 1 | .706** | .718** | .610** | 0.398 | .449* | -.577** |
| MBN | .498* | -0.061 | .706** | 1 | .545* | 0.386 | .785** | .823** | -.605** |
| HFON | .724** | 0.435 | .718** | .545* | 1 | .952** | .445* | 0.384 | -.784** |
| HFOC | .681** | .519* | .610** | 0.386 | .952** | 1 | 0.346 | 0.275 | -.738** |
| LFON | .614** | -0.102 | 0.398 | .785** | .445* | 0.346 | 1 | .951** | -.583** |
| LFOC | .481* | -0.126 | .449* | .823** | 0.384 | 0.275 | .951** | 1 | -.446* |
| pH | -.764** | -0.263 | -.577** | -.605** | -.784** | -.738** | -.583** | -.446* | 1 |

**: P<0.01, *: P<0.05.
