# Supplementary material for: Soil organic carbon fraction accumulation and bacterial characteristics in curtilage soil: Effects of land conversion and land use
Source: PLoS One. 2023 Apr 6;18(4):e0283802. doi: 10.1371/journal.pone.0283802 (PMC10079021; doi:10.1371/journal.pone.0283802)
Supplement: Attachment — Submitted filename: PONE-D-22-15913.pdf [file pone.0283802.s008.pdf]

PLOS ONE

SOC fractions accumulation and microbial characteristic in curtilage soil: effects of land conversion as well as land use

--Manuscript Draft--

**Manuscript Number:**	PONE-D-22-15913
**Article Type:**	Research Article
**Full Title:**	SOC fractions accumulation and microbial characteristic in curtilage soil: effects of land conversion as well as land use
**Short Title:**	SOC fractions accumulation and microbial characteristic in curtilage soil
**Corresponding Author:**	Dongxu Cui<br>Shandong Jianzhu University<br>Jinan, Shandong Province CHINA
**Keywords:**	bacterial composition, curtilage soil, dissolved organic carbon, microbial biomass carbon, soil conversion, soil organic matter
**Abstract:**	A large area of curtilage is converting into cropland or grassland with the rural revitalization strategy in China, while the effects of land conversion on soil nutrition and microbial activities are few studied. This study represents the first attempt to present the composition features of soil organic carbon (SOC) fractions and bacterial communities in rural curtilage soil and the converted cropland and grassland in contrast to cropland and grassland soil. Contents of light fraction (LF) and heavy fraction (HF) of OC, dissolved organic carbon (DOC) and microbial biomass carbon (MBC) were determined, and microbial high-throughput analysis was also conducted. It presented significantly low contents of OC fractions in curtilage soil, similar contents of both HFOC and DOC in cropland and grassland, and increased LFOC content from curtilage soil to grassland and to cropland. Proteobacteria, Actinobacteria and Chloroflexi were predominant in cropland, grassland and curtilage soil, respectively, and cropland showed notably high bacterial richness and diversity. In the perspective of land conversion, converted cropland and grassland showed the increase in LFOC, the same level of HFOC and DOC, and a significant decrease in MBC compared with curtilage soil. The effect of land conversion on microbial composition was more remarkable than that of land-use differences. Further analysis showed that the abundant population of Actinobacteria and Micrococcaceae , and the low contents of MBC indicated microbial state of “hungry” in the converted soil, while the high MBC content, Acidobacteria proportion, and relative abundance of functional genes in the biosynthesis of fatty acid and lipid indicated the microbial state of “fat” in cropland. This study contributes to the development of soil fertility, and improves the understanding and efficient use on rural curtilage soil.
**Order of Authors:**	Qingqing Cao, Ph. D
	Bing Liu
	Jinhang Wu, master
	Xu Zhang, Ph. D
	Wen Ma
	Dongxu Cui
**Additional Information:**	
**Question**	**Response**
**Financial Disclosure**<br><br>Enter a financial disclosure statement that describes the sources of funding for the work included in this submission. Review the submission guidelines for detailed	Yes. This study is supported by the National Key Research and Development Plan “Research on the spatial optimization and layout of rural and communities” (NO. 2019YFD1100801), recieved by Dongxu Cui. Prof Cui participates in the study design, data analysis and manuscript writing.

requirements. View published research articles from *PLOS ONE* for specific examples.<br><br>This statement is required for submission and **will appear in the published article** if the submission is accepted. Please make sure it is accurate.<br><br>**Unfunded studies**<br>Enter: *The author(s) received no specific funding for this work.*<br><br>**Funded studies**<br>Enter a statement with the following details:<br>• Initials of the authors who received each award<br>• Grant numbers awarded to each author<br>• The full name of each funder<br>• URL of each funder website<br>• Did the sponsors or funders play any role in the study design, data collection and analysis, decision to publish, or preparation of the manuscript?<br>• **NO** - Include this sentence at the end of your statement: *The funders had no role in study design, data collection and analysis, decision to publish, or preparation of the manuscript.*<br>• **YES** - Specify the role(s) played.<br><br>* typeset	
**Competing Interests**<br><br>Use the instructions below to enter a competing interest statement for this submission. On behalf of all authors, disclose any competing interests that could be perceived to bias this work—acknowledging all financial support and any other relevant financial or non-financial competing interests.<br><br>This statement is required for submission and **will appear in the published article** if the submission is accepted. Please make sure it is accurate and that any funding sources listed in your Funding Information later in the submission form are also declared in your Financial Disclosure	NO authors have competing interests

statement.<br><br>View published research articles from *PLOS ONE* for specific examples.<br><br>**NO authors have competing interests**<br><br>Enter: *The authors have declared that no competing interests exist.*<br><br>**Authors with competing interests**<br><br>Enter competing interest details beginning with this statement:<br><br>*I have read the journal's policy and the authors of this manuscript have the following competing interests: [insert competing interests here]*<br><br>* typeset	
**Ethics Statement**<br><br>Enter an ethics statement for this submission. This statement is required if the study involved:<br><br>• Human participants<br>• Human specimens or tissue<br>• Vertebrate animals or cephalopods<br>• Vertebrate embryos or tissues<br>• Field research<br><br>Write "N/A" if the submission does not require an ethics statement.<br><br>General guidance is provided below. Consult the submission guidelines for detailed instructions. **Make sure that all information entered here is included in the Methods section of the manuscript.**	N/A

**Format for specific study types**<br><br>**Human Subject Research (involving human participants and/or tissue)**<br>• Give the name of the institutional review board or ethics committee that approved the study<br>• Include the approval number and/or a statement indicating approval of this research<br>• Indicate the form of consent obtained (written/oral) or the reason that consent was not obtained (e.g. the data were analyzed anonymously)<br><br>**Animal Research (involving vertebrate animals, embryos or tissues)**<br>• Provide the name of the Institutional Animal Care and Use Committee (IACUC) or other relevant ethics board that reviewed the study protocol, and indicate whether they approved this research or granted a formal waiver of ethical approval<br>• Include an approval number if one was obtained<br>• If the study involved *non-human primates*, add *additional details* about animal welfare and steps taken to ameliorate suffering<br>• If anesthesia, euthanasia, or any kind of animal sacrifice is part of the study, include briefly which substances and/or methods were applied<br><br>**Field Research**<br><br>Include the following details if this study involves the collection of plant, animal, or other materials from a natural setting:<br>• Field permit number<br>• Name of the institution or relevant body that granted permission	
**Data Availability**<br><br>Authors are required to make all data underlying the findings described fully available, without restriction, and from the time of publication. PLOS allows rare exceptions to address legal and ethical concerns. See the PLOS Data Policy and FAQ for detailed information.	Yes - all data are fully available without restriction

A Data Availability Statement describing where the data can be found is required at submission. Your answers to this question constitute the Data Availability Statement and **will be published in the article**, if accepted.<br><br>**Important:** Stating 'data available on request from the author' is not sufficient. If your data are only available upon request, select 'No' for the first question and explain your exceptional situation in the text box.<br><br>Do the authors confirm that all data underlying the findings described in their manuscript are fully available without restriction?	
**Describe where the data may be found in full sentences. If you are copying our sample text, replace any instances of XXX with the appropriate details.**<br><br>• If the data are **held or will be held in a public repository**, include URLs, accession numbers or DOIs. If this information will only be available after acceptance, indicate this by ticking the box below. For example: *All XXX files are available from the XXX database (accession number(s) XXX, XXX.)*.<br>• If the data are all contained **within the manuscript and/or Supporting Information files**, enter the following: *All relevant data are within the manuscript and its Supporting Information files.*<br>• If neither of these applies but you are able to provide **details of access elsewhere**, with or without limitations, please do so. For example:<br><br>*Data cannot be shared publicly because of [XXX]. Data are available from the XXX Institutional Data Access / Ethics Committee (contact via XXX) for researchers who meet the criteria for access to confidential data.*<br><br>*The data underlying the results presented in the study are available from (include the name of the third party*	The data underlying the results presented in the study are available from https://www.genescloud.cn/analysisProcess/projectOverview

and contact information or URL).<br>• This text is appropriate if the data are owned by a third party and authors do not have permission to share the data.<br><br>* typeset	
Additional data availability information:	Tick here if the URLs/accession numbers/DOIs will be available only after acceptance of the manuscript for publication so that we can ensure their inclusion before publication.

Powered by Editorial Manager® and ProduXion Manager® from Aries Systems Corporation

Manuscript
Click here to access/download;Manuscript;manuscript for plos one.docx

~~SOC~~ fractions accumulation and microbial characteristic in curtilage soil: effects of land conversion as well as land use

Qingqing Cao[1#], Bing Liu[2#], Jinhang Wu[1], Xu Zhang[1], Wen Ma[3], Dongxu Cui[1]*

[1] School of Architecture and Urban Planning, Shandong Jianzhu University, Jinan, China;

[2] School of Municipal and Environmental Engineering, Shandong Jianzhu University, Jinan, China;

[3] College of Chemistry and Chemical Engineering, Qilu Normal University, Jinan, China

[#]First author: Qingqing Cao and Bing Liu

*Corresponding author: Dongxu Cui

School of Architecture and Urban Planning,

Shandong Jianzhu University, Jinan, 250101, China;

Tel: +86 13708920109

E-mail: cdx0109@sdjzu.edu.cn

Abstract

A large area of curtilage is converting into cropland or grassland with the rural revitalization strategy in China, while the effects of land conversion on soil nutrition and microbial activities are few studied. This study represents the first attempt to present the composition features of soil organic carbon (SOC) fractions and bacterial communities in rural curtilage soil and the converted cropland and grassland in contrast to cropland and grassland soil. Contents of light fraction (LF) and heavy fraction (HF) of OC, dissolved organic carbon (DOC) and microbial biomass carbon (MBC) were determined, and microbial high-throughput analysis was also conducted. It presented significantly low contents of OC fractions in curtilage soil, similar contents of both HFOC and DOC in cropland and grassland, and increased LFOC content from curtilage soil to grassland and to cropland. Proteobacteria, Actinobacteria and Chloroflexi were predominant in cropland, grassland and curtilage soil, respectively, and cropland showed notably high bacterial richness and diversity. In the perspective of land conversion, converted cropland and grassland showed the increase in LFOC, the same level of HFOC and DOC, and a significant decrease in MBC compared with curtilage soil. The effect of land conversion on microbial composition was more remarkable than that of land-use differences. Further analysis showed that the abundant population of Actinobacteria and *Micrococcaceae*, and the low contents of MBC indicated microbial state of “hungry” in the converted soil, while the high MBC content, Acidobacteria proportion, and relative abundance of functional genes in the biosynthesis of fatty acid and lipid indicated the microbial state of “fat” in cropland. This study contributes to the development of soil fertility, and improves the understanding and efficient use on rural curtilage soil.

Keywords: bacterial composition, curtilage soil, dissolved organic carbon, microbial biomass carbon, soil conversion, soil organic matter

1. Introduction

Rural revitalization strategy is firstly reported by the 19th National Congress of the Communist Party of China, it aims to improve the natural, social and economic levels in rural areas (Zhang 2019; Zuo 2019). In this process, numerous villages are integrated into communities based on the developing demand of agricultural modernization (Jiang and Wang 2013). Meanwhile, rural land use has great changes, large areas of rural curtilage soil (RCS) are converted into cropland and grassland. Preliminary visit and investigation showed that the average crop yield is 15742 kg per hectares in common cropland of Shandong Province, while that is only 9895 kg per hectares in the converted cropland from curtilage for five years. Moreover, the farmers reflect that fertilization and deep ploughing cannot improve the crop production notably. It indicates that soil fertility and nutrition could be largely affected by the curtilage conversion, while the detailed effects are not clear. Furthermore, soil organic matter (SOM) and microbes, the key indicators of soil fertility and nutrition, may be the main reasons for the low soil quality and crop yield in the converted soils (Humbert et al. 2020), however, the current research on RCS is very insufficient.

The SOM mainly refers to a kind of complex and relatively stable polymer organic compounds that formed by microbiological degradation, it mainly composes of sugar, organic acids, cellulose, hemicellulose, lignin, lipid and protein and so on (Karhu et al. 2010; Man et al. 2021; Dong et al. 2020). Moreover, it is also the nutrient reservoir that profoundly affects the soil physical, chemical and biological properties (Karhu et al. 2010; Angst et al. 2021). Previous study indicated that 1% of the organic carbon is equivalent to 270 kg per hectares of nutrients in the soil (Zhang and He 2004). Other studies also showed that the soil fertility capacity would reduce by 14% when its organic matter content descends from 2% to 1.5% (Solttsova and Danilovic 2011). Thus, the study on organic matter of RCS is very meaningful. SOM is mainly from the input of microbial activities, animals and plants, and anthropogenic activities (Mccorkle et al. 2016). Such as the input of crop roots, straw returning to the

field, the input of human manure, household garbage and sewage, and residuals and metabolite of soil microorganisms, animals and plants (Aukes and Schiff 2021; Li et al. 2021; Fu et al. 2021). However, increasing the SOM cannot directly increase soil fertility, the microbial decomposition, biosynthesis and metabolism process of SOM is the most important link during the SOM transformation and reuse by crops (Kauer et al. 2019; Aytenew and Bore 2020).

According to the diversity and complexity of its sources, ~~OM~~ has different morphology and classification (Dong et al. 2020). When OM is input into the soil, most of it would be converted to carbon dioxide by microbial decomposition (Song et al. 2021). Only a small fraction of the OM can remain in the soil, among which, undecomposed or partly decomposed residues of animals, plants and microbes account for about 1%~5%, they are previously studied and known as light fraction organic matter (LFOM) (Rui et al. 2016). Soil microorganism accounts for 3%~8% of the SOM (Xu et al. 2012). The dissolved organic matter (DOM) is the main form of OC transfer among plant, microorganism and soil, such as small-molecule of polysaccharides and organic acids (Li et al. 2020; Aukes and Schiff 2021). It commonly accounts for 3%~8% of SOM, participates in microbial synthesis and metabolism (Chen et al. 2016). The above-mentioned OM fractions are the dominant components of active organic matter, carbon and nitrogen contents of which are closely and directly related to the soil microbial activities. In addition, with complex chemical structure and stable physicochemical properties, heavy fraction organic matter (HFOM) accounts for more than 60% of the remained OM, being the major component of SOM reservoir pool (Rondon et al. 2021). Thus, characteristic of SOM fractions and microbial activities affected by land use or land conversion may have significant differences, thus impact soil fertility and nutrition. However, previous researches on SOM and microorganism mostly focus on the land use changes from returning farmland to grassland, wetland or forest, while few relevant research involve in the RCS, or converted cropland and grassland from curtilage.

With the support of China National Key R&D Program during the 13th Five-year Plan Period “Research on the spatial optimization and layout of rural and

communities", we conducted the study on SOC fractions accumulation and microbial characteristic in soil of rural curtilage, cropland, grassland, converted cropland (CCS) and converted grassland (CGS) from curtilage. This study aimed to: (1) present the contents and distribution of SOM fractions, (2) show the bacterial composition and functional differences, (3) analyze the inter connections between OC or ON fractions and bacterial communities, (4) expound the carbon and nitrogen accumulation potential in the five land types. This study contributes to the development of soil fertility, and improves the understanding and efficient use on rural curtilage soil.

2. Materials and Methods

2.1 Study area and field sampling

The Yellow River town is in the northern part of Zhangqiu district, Jinan city of Shandong Province, China. Its northwest side is next to the Yellow River; thus, the soils and human activities were deeply affected by the Yellow River. For this study, we investigated the Yellow River town and confirmed the research area (36°57′45″-36°59′17″N, 117°15′21″108°31′59″-117°18′10″E) (Fig 1). The soil of RCS, cropland, grassland, CGS and CCS were sampled. Among which, CGS and CGS were the soils that has been converted from rural curtilage for five years. The area is located in a temperate monsoon climate during the same season. The average annual temperature is 12.8□, and the average annual precipitation is 600.8 mm. The frost-free period is 192 days, and the main soil type is tidal soil (Qiu et al. 2015).

The soil sampling was conducted in May 2021 by a soil auger. One mixed soil sample at each site was collected based on the five-point sampling method in a 0.5 m × 0.5 m quadrat, and the coordinates and altitude information were recorded by a global positioning system instrument. Four soil samples were taken for each land type. Every soil sample was divided into three parts, one part of soil was stored at -20 °C for microbial composition analysis, one part was stored at 4 °C for analysis of microbial biomass carbon and nitrogen (MBC & MBN) and DOM, and the other part was stored at room temperature for analysis of LFOM and HFOM. Totally, twenty surface soil samples (0-10 cm) were collected. The detailed distribution of sampling

sites was showed in Fig 1.

2.2. The analysis of LFOM and HFOM

Soil samples were air-dried, ground, and sieved through a 0.9 mm sieve after grinding, and 10.00 g of each soil sample was weighted and mixed with 40 mL of 1.85 $g{\cdot}mL^{-1}$ Sodium Iodide solution for the soil stratification of LF and HF. Then, 0.043 mm brass sieves and calcium chloride solution were employed for the separation and washing of LF and HF respectively. After that, diluted hydrochloric acid (1:10 of volume) was employed to remove the inorganic carbon. Finally, distilled water was used to wash away the chloride ions. The oven-dried HF and LF were determined by an element analyzer (Vario EL III, Elementar Analysensysteme, Germany) (Dong et al. 2020).

2.3. The analysis of DOM, MBC and MBN

The chloroform fumigation method was conducted to all the soil samples to kill the microbes (Xu et al. 2012). Concretely, 10.00 g fresh soil, sodium hydrate solution, and 20 mL of chloroform were put into a drying cabinet, a vacuum pump was implemented to boil the chloroform absolutely. Then, chloroform was removed and the drying cabinet was stand for a night to kill all the microbes, and the drying cabinet was vacuumized to clear the gasified chloroform. Meanwhile, another part of weighted fresh soil and sodium hydrate solution were placed into a drying cabinet as control. The DOM in the fumigated soil and control soil were extracted with 2 M potassium chloride (KCL) solution at 1:5 ratio. The carbon and nitrogen contents in all the leaching solution were determined by a carbon/nitrogen analyzer (ANALYTIKJENA MULTI N/C 3100, Elementar Analysensysteme, Germany), and the contents of MBC and MBN were the carbon or nitrogen contents in leaching solution of fumigated soil subtracting those of controlled soil respectively.

In addition, 10.00 g fresh soil was extracted by 2 M of KCl solution with 1:5 ratio. The carbon/nitrogen contents in leaching solution were examined and the DOC and DON contents were calculated (Han et al. 2021). Thus, soil organic carbon (SOC) can be calculated by adding the contents of DOC, LFOC and HFOC together.

Moreover, soil pH was determined by a pH meter.

2.4. Extraction and Illumina sequencing

Soil was freeze-drying and sieved, and DNA from each weighted soil samples were extracted by the DNA isolation kit (MO-BIO Laboratories, Carlsbad, CA, USA) following the manufacturer's guidelines. Then, the DNA was verified by 2% agarose gel electrophoresis, and determined by a Nanodrop ND-1000 UV-Vis spectrophotometer (Thermo Fisher Scientific, Waltham, MA, USA) (Fang et al., 2019). Moreover, the DNA were diluted to 1 ng/μL for further analysis. Polymerase chain reaction of V3-V4 region based on the primer (F: ACTCCTACGGGAGGCAGCA and R: TCGGACTACHVGGGTWTCTAAT) was conducted, and Phusion High-Fidelity PCR Master Mix with GC Buffer and efficient high-fidelity enzyme were employed to make sure amplification accuracy. Furthermore, 2% agarose gel electrophoresis and the gel recovery kit (Qiagen Gel Extraction Kit, Germany) were used to examine and recover the polymerase chain reaction products (Nithya et al. 2015). The DNA sequencing library was produced, quantified and detected, and then it was paired-end sequenced on the NovaSeq6000 (Illumina, San Diego, CA, USA) platform (Dong et al. 2020).

2.5. Statistical analysis

The mean analysis and one-way analysis of variance were conducted to analyze the mean values and significant difference of C and N fractions in the five soil types. Pearson correlation analysis was used to analyze the probably connections among the C fractions, N fractions, pH and bacterial composition. Principal coordinates analysis based on the Bray-Curtis distance was implemented according to bacterial composition. In addition, bacterial function prediction was analyzed with Phylogenetic Investigation of Communities by Reconstruction of Unobserved States (PICRUSt) based on the data library. The SPSS v22.0, QIIME2 (2019.4) and R Studio were used to analyzing the data and Origin v2018, Adobe Illustrator CS5, and ArcGIS v10.2.2 were used to draw figures.

3. Results and Discussion

3.1. Carbon and nitrogen fractions in different soil types

The OM fractions between cropland and grassland showed different characteristic including the content and composition, which indicated similar regularity with the previous research (Song et al. 2018). As shown in Fig 2, DOC contents in grassland, cropland and CGS were significantly higher than in CCS or RCS. The contents of DOC were sorted as: RCS (8.36 $mg{\cdot}kg^{-1}$) < CCS (11.38 $mg{\cdot}kg^{-1}$) < CGS (16.49 $mg{\cdot}kg^{-1}$) < cropland (17.95 $mg{\cdot}kg^{-1}$) < grassland (18.19 $mg{\cdot}kg^{-1}$), which indicating that 1) RCS had the lowest DOC content; 2) DOC contents in grassland were higher than in cropland, and not changed by the construction land conversion. Previous studies also indicated the high DOC contents in grassland than cropland (Jones et al. 2014), and abundant microbial degradation activities in unfavorable and barren grassland environment probably play a decisive role (Humbert et al. 2020). The lowest DOC in RCS may indicate the low microbial abundance and activities.

MBC and MBN were reportedly sensitive to different land types, which can also indicate the microbial abundance (Chen et al. 2016; Pujia et al. 2017). MBC contents were sorted as: CGS (23.46 $mg{\cdot}kg^{-1}$) < CCS (40.67 $mg{\cdot}kg^{-1}$) < RCS (55.80 $mg{\cdot}kg^{-1}$) < grassland (75.73 $mg{\cdot}kg^{-1}$) < cropland (111.86 $mg{\cdot}kg^{-1}$; Fig 2). MBC contents presented increased trend from RCS, to grassland, and to cropland, suggesting the highest and lowest microbial abundance of cropland and RCS respectively, corroborated by the Chao1 Index and Observed Species Index (Table 1). Quadros et al. (2016) showed the low microbial diversity and biomass in post-mined soil. Our result suggested that land construction activities can seriously damage the habitat of soil microorganisms. Interestingly, the converted soil showed notably lower MBC contents than RCS, thus the land conversion increased the microbial richness and diversity but reduced the MBC contents. In addition, Chao1 Index and Observed Species Index were notably higher in cropland than CCS while lower in grassland than in CGS, indicating the reverse effect of land conversion on cropland and grassland. The finding probably affected by bacterial composition difference (Table

1). Shannon Index, as the bacterial diversity index, also showed the same trend.

Table 1. The mean values of bacterial richness and diversity indexes.

Land use types	Chao1 Index	Observed Species Index	Shannon Index
Rural curtilage	5232.8**a**[1]	4335.3**a**	10.6**a**
Grassland	6711.0**b**	5631.1**b**	10.9**ab**
Cropland	8749.4**c**	6913.5**c**	11.6**c**
CGS	7450.2**b**	5932.8**b**	11.3**bc**
CCS	6610.2**b**	5208.8**ab**	10.8**ab**

[1] The same letters in each column indicated non-significant difference at α=0.05 level (Duncan test).

As one component, LFOM could be degraded, absorbed and transferred by microbes (Dong et al. 2020). LFOC and LFON presented similar distribution pattern, which was significantly the lowest (3.91 and 0.14 $mg \cdot kg^{-1}$ respectively) in RCS and the highest (15.08 and 0.86 $mg \cdot kg^{-1}$ respectively) in cropland. Plant residue returning to the field provided substantial source for LFOM accumulation (Lei et al., 2013), and it presented insignificant difference under the effect of land conversion. HFOM was the dominant component of soil carbon pool or nitrogen pool due to its stable property (Tan et al. 2007). HFOC and HFON showed significantly higher accumulation in grassland and cropland than in other soil. Thus HFOM distribution was notably affected by both land-use difference and land conversion. Li et al. (2021) showed that straw retention can promote the accumulation of stable organic carbon, while this result was inapplicable when faced with curtilage conversion. Wang et al. (2020) showed the reverse distribution pattern of MBC and HFOC, thus microbial richness and composition probably important factors affecting the stable C accumulation.

3.2. Bacterial composition in different soil types

Proteobacteria, Actinobacteria, Acidobacteria, Chloroflexi, Firmicutes, Gemmatimonadetes, and Bacteroidetes were the dominant population in the studied soil (Fig 3). Different land types exhibited specificity in the composition and quantity of microbial communities. With the proportions of 35.18% and 18.13%, cropland presented significantly higher Proteobacteria and Acidobacteria than other soil types. Actinobacteria was remarkedly higher in CGS (31.13%) and CCS (31.85%) than grassland (26.58%) or cropland (20.91%), and it was notably the lowest in RCS (14.71%). Based on the degradation function on long chain n-alkanes and other

organic matters of Actinobacteria (Pasquale et al. 2012), RCS presented low OM degradation capacity, and converted soil showed the high degradation capacity. In family level, *Subgroup 6*, *Micrococcaceae*, *Nitrosomonadaceae*, *KD4-96*, and *Gemmatimonadaceae* were the identified abundant families (Fig 3). Concretely, cropland had significantly high proportion of *Subgroup 6* (10.66%), which may affected by the abundant active OC and soil pH (Wu et al. 2021). The CGS, CCS and grassland presented notably high proportion of *Micrococcaceae*, based on its degradation capacities, the finding suggested high carbon mineralization in the soil types.

The principal coordinates analysis of all the soil types indicated similar cluster of CGS and CCS, a cluster of RCS samples and a cluster of cropland samples (Fig S2), the result showed the significant effect of land conversion on microbial composition, which was higher than the land-use effects between cropland and grassland based on the similar bacterial composition feature and closely internal relations between CGS and CCS. Roadside, the main growth area of grass in countryside, was easily affected by human activities. The scattered distribution of the four grassland samples showed notably differences of microbial composition, which probably be attribute to the human activities.

Result of PICRUSt2 showed that biosynthesis was the major bacterial activities, such as the biosynthesis of amino acid, nucleoside and nucleotide (Fig 4a). Mostly, microbial relative abundance of functional genes in RCS were significantly high in the biosynthesis of nucleoside and nucleotide, amino acid, carbohydrate and cell structure, and generation of precursor metabolite and energy, while were notably low in the degradation, utilization, and assimilation (Table S1). It may suggest that soil microbes proliferated fleetly after released from the perennial high pressure. Concretely, with the average relative abundance of 4179, biosynthesis of cytochrome c in aerobic respiration was the dominant bacterial activities (Qu et al. 2015). Thereinto, bacterial composition and contribution to the cytochrome c biosynthesis in family level was presented in Fig 5. It showed that Proteobacteria, Actinobacteria, Acidobacteria and Chloroflexi were the main bacterial phyla and *Subgroup 6*,

Nitrosomonadaceae, *KD4-96*, *Gemmatimonadaceae*, and *Bacillaceae* were the dominant families that participating in cytochrome c biosynthesis, which suggested the rapid growth in abundance of aerobic microorganisms.

3.3. Connections between OM fractions and bacterial communities

The OM fractions and microbial phyla proportions were used as dependent variables and covariants, neural network was conducted to analyze the microbial importance to OM fractions (Fig S3). Results showed that Planctomycetes had important and positive effect the accumulation of DOC and HFOC. As previous reported, Planctomycetes was significantly affected by soil management history and environmental heterogeneity, which can also notably affect the HFOC content (Buckley et al. 2006; Tan et al. 2007). Planctomycetes also had notably associations with allochthonous and autochthonous tyrosine-like components of DOM. We suggested that Planctomycetes probably participated in the transformation and production of DOM and HFOM. Thus, the high proportion of Planctomycetes implemented non-negligible effect on the high contents of DOC, DON, HFOC, and HFON in grassland. Moreover, Acidobacteria presented the largest effect on MBC and MBN. Xu et al. (2021) showed dominant proportion of Acidobacteria in low-nutrient habitat and Chaves et al. (2019) showed its degradation capacity on complex OM, such as hemicellulose. Thus, through the degradation of biological residues, Acidobacteria promoted the OM transformation and accumulation of MBC and MBN. Thus, the notably low proportions of Acidobacteria in the CGS and CCS can explain the low contents of MBC and MBN. LFOC and LFON offered numerous nutrition for heterotrophic microorganism, thus they were interrelated with the microbes, and presented positive and significant associations with bacterial richness and diversity indexes.

Though Actinobacteria was closely related with OC degradation, proportion of Actinobacteria showed no significant relationship with contents of any C or N fraction. Interestingly, Actinobacteria was positively associated with the DOC proportion (DOC: SOC; R^2=0.404, p=0.003) and negatively related with MBC

proportion (MBC: SOC; R^2=0.255, p=0.023). The result showed that Actinobacteria promoted the MBC degradation and DOC production, especially in CCS and CGS. As one abundant family of Actinobacteria, *Micrococcaceae* can resist adverse soil environment by degradation activities (Finn et al. 2021), its remarkably connection with MBC content indicated the substantial depletion of microbial-derived C. As a result, microorganism in the converted soil was in the state of "hungry". However, in cropland, the relative abundance of functional genes in the biosynthesis of fatty acid and lipid was notably high (Fig 5), and it was positively correlated with the content of MBC and MBN (Fig 4b). The finding suggested that microorganism in cropland was in the state of "fat" with high contents of C and N fractions. As active OC, DOC, LFOC and MBC were significantly and positively correlated, and they were also significantly related to Chao1 Index, Observed Species Index and Shannon Index (Table S2). Thus, they provided plenty nutrition and energy for microbial developing and metabolism. As the undecomposed OC and major degradation products, LFOC and DOC can be significantly affected by MBC and microbial composition (Dong et al. 2020).

The soil pH value, as the most important soil physicochemical parameter, could directly interfere with the microbial community, or indirectly affect the microbial community by affecting the composition of SOM. The pH values were sorted as: RCS (9.04) > CCS (8.91) > CGS (8.59) > grassland (8.28) > cropland (8.17), indicating that pH presented decreased trend after curtilage conversion (Fig S4), but still in an alkaline state. Previous study showed that high pH and alkalescent soil habitat were prone to accumulate organic matter (Gudmundsso et al. 2004), while the extremely significant and negative relations between the C and N fractions and pH suggested that alkaline soil was not always beneficial to OM accumulation and store, thus, negatively affected the microbial richness and diversity (Table S3). On the other side, plant roots can secrete large amounts of organic acids (Fang et al. 2019), which contributed to the decrease in pH values from RCS to converted soil. In return, soil bacterial composition can be changed with the environmental adaptability. However, soil pH had little effects on the relative abundance of bacterial functional genes based

on their non-significant relation (Fig 6).

HFOC and HFON also had no notable association with the main functional genes, indicating that they were not easily affected by bacterial activities. Moreover, DOC, LFOC, and LFON had closely connection with most functional genes, among which, they had significantly and negatively correlations with most functional genes of biosynthesis and generation of precursor, metabolite and energy (such as electron transfer). The finding might suggest that bacterial functional activities can be regulated by currant contents of the C and N fractions (Finn et al. 2021). In addition, the positive associations between DOC, LFOC, LFON and bacterial degradation activities suggested that they had numerous microbial degraded products.

3.4 Carbon and nitrogen accumulation potential

The proportions of each C fractions were determined by compared with the SOC. And the proportion of stable OC can be showed by subtracting contents of MBC, LFOC and DOC from SOC contents (Fig 6). In the view of land-use difference, SOC was significantly higher in cropland and grassland than in RCS, with the highest value of 258.39 mg·kg^{-1} in grassland. This result was similar with the previous reports (Ding et al. 2020), which indicated the advantage of microbial residue C accumulation in grassland. In this study, grassland presented significantly higher proportion and content of stable OC but lower contents of active C fractions than cropland, which may promoted to the transformation of active C fractions into stable OC. While we found that the low contents of active C fractions in the converted soil were the major disparity compared with grassland or cropland, especially the MBC contents. Thus relevant strategies on enhancing MBC content in the converted soil can be a shortcut for encouraging soil carbon cycle and accumulation.

For N fractions, grassland showed significantly higher content and proportion of stable OC and DON, while cropland presented the notably higher MBN among the land-use types. Soil management and fertilization, as the largest difference between grassland and cropland, increased microbial biomass, enzymatic activities and further the ON utilize and consumption (Guo et al. 2011), thus resulting in the low content of

total ON in cropland (19.08 mg·kg^{-1}) than grassland (25.57 mg·kg^{-1}). The notably high DON contents in grassland indicated low requirement in the growth of grass than crop.

The value of C: N is a good indicator for C and N mineralization in soil (Roberts et al. 2009). Thus, the lowest C: N value in grassland (10.10) suggested the low C and N mineralization rate while the notably high values in CGS (14.86) and CCS (15.34) indicated the high mineralization rate. The values of MBC: LFOC and values of MBC: DOC can be regarded as the amount of unit MBC that degraded the total LFOC or produced the total DOC. The values presented notably differences among the five land-use types, and they were related at a exponential function with R^2 of 0.736. It indicated potential rule between the consumption of LFOC and the production of DOC, which was negatively regulated by Actinobacteria based on their significant differences.

4. Conclusions

In this study, the C and N fractions were determined, microbial composition and function gene abundance were analyzed, and connections between OM fractions and microbial communities were discussed among the five land-use types. For soil from rural curtilage (RCS), cropland, and grassland, it presented significantly low OM fractions in RCS, similar accumulation of HFOM and DOC in cropland and grassland, and increased LFOM and microbial-OM from RCS to grassland and to cropland. Proteobacteria, Actinobacteria and Chloroflexi were predominant in cropland and grassland and RCS respectively, and cropland showed high bacterial richness and diversity. In the perspective of land conversion, cropland (CCS) and grassland (CGS) converted from curtilage showed the increase in LFOM and MBN, the same level of HFOM and DOM, and a significant decrease in MBC compared with RCS. The effect of land conversion on microbial composition was more significant than that of land-use differences based on the principal coordinates analysis. The abundant population of Actinobacteria and *Micrococcaceae*, and the low contents of MBC indicated microbial state of “hungry” in the converted soil, while the high MBC content,

Acidobacteria proportion, and relative abundance of functional genes in the biosynthesis of fatty acid and lipid indicated the microbial state of “fat” in cropland. In addition, grassland presented high carbon accumulation potential based on the high SOC content and low value of C: N, while converted soil had high C minerilization. Therefore, the above-mentioned results contributed to the poor growth state of crop or grass in converted soil but vigorously growth of wheat in cropland.

This study represents the first attempt to present the composition features of SOM and bacterial communities in rural curtilage soil, as well as their possible changes after converted to cropland and grassland. It contributes to the increasing in agricultural yield and soil fertility, and improves the understanding and efficient use on curtilage converted land. Meanwhile, further attempts will be conducted on how to enrich the MBC content and bacterial functions in the converted soil effectively.

Acknowledgments

We thank Shanghai Personal Biotechnology Co., Ltd. (Shanghai, China) for microbial Illumina Sequencing. We also thank Dr. Junyu Dong for the revision. This study was supported by the National Key Research and Development Plan "Research on the spatial optimization and layout of rural and communities" (NO. 2019YFD1100801).

Conflicts of Interest

The authors declare no conflict of interest.

References

Angst G, Pokorn J, Mueller CW, Prater I, Angst S. Soil texture affects the coupling of litter decomposition and soil organic matter formation. Soil Biol Biochem 2021; 159: 108302.

Aukes P, Schiff SL. Composition wheels: visualizing dissolved organic matter using common composition metrics across a variety of Canadian Ecozones. PLoS ONE, 2021; 16(7): e0253972.

Aytenew M, Bore G. Effects of organic amendments on soil fertility and environmental quality: A review. J Plant Sci 2020; 8: 112-119.

Buckley DH, Huangyutitham V, Nelson TA, Rumberger A, Thies JE. Diversity of *planctomycetes* in soil in relation to soil history and environmental heterogeneity. Appl Environ Microbiol 2006; 72: 4522-4531.

Chaves M, Silva G, Rossetto R, Edwards RA, Navarrete AA. Acidobacteria subgroups and their metabolic potential for carbon degradation in sugarcane soil amended with vinasse and nitrogen fertilizers. Front Microbio 2019; 10:1680.

Chen J, Sun X, Li L, Liu X, Zhang B, Zheng J, et al. Change in active microbial community structure, abundance and carbon cycling in an acid rice paddy soil with the addition of biochar. Eur J Soil Sci 2016; 67: 857-867.

Dong J, Quan Q, Zhao D, Li C, Liu L. A combined method for the source apportionment of sediment organic carbon in rivers. Sci Total Environ 2020; 752: 141840.

Dong L, Li J, Sun J, Yang C. Soil degradation influences soil bacterial and fungal community diversity in overgrazed alpine meadows of the Qinghai-tibet Plateau. Sci Rep 2021; 11: 11538.

Fang J, Zhao R, Cao Q, Quan Q, Sun R, Liu J. Effects of emergent aquatic plants on nitrogen transformation processes and related microorganisms in a constructed wetland in northern China. Plant Soil 2019; 443: 473-492.

Finn DR, Maldonado J, Martini FD, Yu J, Garcia-Pichel F. Agricultural practices drive biological loads, seasonal patterns and potential pathogens in the aerobiome of a mixed-land-use dryland. Sci Total Environ 2021; 798: 149239.

Fu L, Xi M, Nicholaus R, Wang Z, Wang X, Kong F, et al. Behaviors and biochemical responses of macroinvertebrate Corbicula fluminea to polystyrene microplastics. Sci Total Environ 2022; 813: 152617.

Guo P, Wang C, Jia Y, Wang Q, Han G, Tian X. Responses of soil microbial biomass

and enzymatic activities to fertilizations of mixed inorganic and organic nitrogen at a subtropical forest in East China. Plant Soil 2011; 338: 355-366.

Han H, Hwang J, Kim G. Characterizing the origins of dissolved organic carbon in coastal seawater using stable carbon isotope and light absorption characteristics. Biogeosciences 2021; 18: 1793-1801.

Humbert G, Parr TB, Jeanneau L, Dupas R, Jaffrezic A. Agricultural practices and hydrologic conditions shape the temporal pattern of soil and stream water dissolved organic matter. Ecosystems 2020; 23: 1325-1343.

Jiang HM, Wang ZH. Empirical analysis on the relationship among industrialization, urbanization and agricultural modernization in Jilin Province. Sci Geogr Sin 2012; 32: 591-595.

Jones DL, Simfukwe P, Hill PW, Mills RTE, Emmett BA. Evaluation of Dissolved Organic Carbon as a Soil Quality Indicator in National Monitoring Schemes. PLoS ONE. 2014; 9(3): e90882

Karhu K, Fritze H, Tuomi M, Vanhala P, Spetz P, Kitunen V, et al. Temperature sensitivity of organic matter decomposition in two boreal forest soil profiles. Soil Biol Biochem 2010; 42: 72-82.

Kauer K, Astover A, Viiralt R, Raave H, Ktterer T. Evolution of soil organic carbon in a carbonaceous glacial till as an effect of crop and fertility management over 50 years in a field experiment. Agr Ecosyst Environ 2019; 283: 106562.

Lei D, Shangguan ZP, Sandr S, Ben BL. Changes in soil carbon and nitrogen following land abandonment of farmland on the Loess Plateau, China. PLoS ONE; 2013; 8(8): e71923.

Li C, Wang X, Qin M. Spatial variability of soil nutrients in seasonal rivers: a case study from the Guo River Basin, China. PLoS ONE, 2021; 16(3): e0248655.

Li J, Jian S, Lane CS, Lu YH, He X, Wang G, et al. Effects of nitrogen fertilization and bioenergy crop type on topsoil organic carbon and total nitrogen contents in middle tennessee usa. PLoS ONE 2020; 15(3): e0230688.

Li YM, Duan Y, Wang GL, Wang AQ, Zhang DM. Straw alters the soil organic carbon composition and microbial community under different tillage practices in a meadow soil in Northeast China. Soil Till Res 2021; 208: 104879.

Man M, Wagner-Riddle C, Dunfield KE, Deen B, Simpson MJ. Long-term crop rotation and different tillage practices alter soil organic matter composition and degradation. Soil Till Res 2021; 209: 104960.

Mccorkle EP, Berhe AA, Hunsaker CT, Johnson DW, Macfarlane KJ, Fogel ML, et al. Tracing the source of soil organic matter eroded from temperate forest catchments using carbon and nitrogen isotopes. Chem Geol 2016; 445: 172-184.

Pasquale CD, Palazzolo E, Piccolo LL, Quatrini P. Degradation of long-chain n-alkanes in soil microcosms by two actinobacteria. J Environ Sci Health 2012; 47, 374-381.

Qu L, Huang Y, Zhu C, Zeng H, Pi E, Rhizobia-inoculation enhances the soybean's tolerance to salt stress. Plant Soil 2015; 400: 209-222.

Quadros P, Zhalnina K, Davis-Richardson AG, Drew JC, Menezes FB, Camargo F, et al. Coal mining practices reduce the microbial biomass, richness and diversity of soil. Appl Soil Ecol 2016; 98: 195-203.

Roberts P, Stockdale R, Khalid M, Iqbal Z, Jones DL. Carbon-to-nitrogen ratio is a poor predictor of low molecular weight organic nitrogen mineralization in soil. Soil Biol Biochem 2009; 41: 1750-1752.

Rondon T, Hernandez RM, Guzman M. Soil organic carbon, physical fractions of the macro-organic matter, and soil stability relationship in lacustrine soils under

banana crop. PLoS ONE, 2021; 16(7): e0254121.

Rui Y, Murphy DV, Wang X, Hoyle FC. Microbial respiration, but not biomass, responded linearly to increasing light fraction organic matter input: consequences for carbon sequestration. Sci Rep 2016; 6: 35496.

Song X, Fang C, Yuan ZQ, Li FM. Long-term growth of alfalfa increased soil organic matter accumulation and nutrient mineralization in a semi-arid environment. Front Environ Sci 2021; 9: 649346.

Song XD, Fan Y, Bing J, Li DC, Zhao YG, Yang JL, et al. The influence of the conversion of grassland to cropland on changes in soil organic carbon and total nitrogen stocks in the Songnen Plain of Northeast China. Catena 2018; 171: 588-601.

Tan Z, Lal R, Owens L, Izaurralde RC. Distribution of light and heavy fractions of soil organic carbon as related to land use and tillage practice. Soil Till Res 2007; 92: 53-59.

Wang H, Wu J, Li G, Yan L. Changes in soil carbon fractions and enzyme activities under different vegetation types of the northern Loess Plateau. Ecol Evol 2020; 10: 12211-12223.

Wu Y, Wang H, Xu N, Li J, Xing J, Zou H. Effects 10 years elevated atmospheric CO_2 on soil bacterial community structure in Sanjiang Plain, Northeastern China. Plant Soil 2021; 471: 73-87.

Xu M, Jian J, Wang J, Zhang Z, Yang G, Han X, et al. Response of root nutrient resorption strategies to rhizosphere soil microbial nutrient utilization along *Robinia pseudoacacia* plantation chronosequence. Forest Ecol Manag 2021; 489: 119053.

Xu X, Thornton PE, Post WM. A global analysis of soil microbial biomass carbon, nitrogen and phosphorus in terrestrial ecosystems: Magnitude, stoichiometry, and pool size. Conference: 97th ESA Annual Convention 2012.

Yang Y, Li T, Wang Y, Dou Y, An S. Linkage between soil ectoenzyme stoichiometry ratios and microbial diversity following the conversion of cropland into grassland. Agr Ecosyst Environ 2021; 314: 107418.

Yu P, Liu S, Han K, Guan S, Zhou D. Conversion of cropland to forage land and grassland increases soil labile carbon and enzyme activities in northeastern China. Agr Ecosyst Environ 2017; 245: 83-91.

Zhang M, He Z. Long-term changes in organic carbon and nutrients of an ultisol under rice cropping in Southeast China. Geoderma 2004; 118: 167-179.

Zhang Y. New thinking on academic journal work under guidance of spirit of the 19th national congress of the communist party of China. J Beijing Polytechnic College 2019.

Zuo X. An ecological discourse analysis of the report of the 19th national congress of the communist party of China. Theor Pract Lang Stud 2019; 9: 1191.

Figure captions

Fig 1. The sampling sites and surrounding environments in this study. (a) indicated the site of Jinan city and Shandong Province, (b) showed the site of Yellow River town, and (c) showed the sampling sites and surroundings in the Yellow River town.

Fig 2. The contents of C and N fractions in the five land-use types. Different letters on bars in each carbon fraction indicated significant differences at α=0.05 level (Duncan test).

Fig 3. The taxonomic composition analysis in levels of phyla and families in the five soil types. (a) bacterial composition in phyla level of all the samples, (b) bacterial composition in phylum level of the five soil types, (c) bacterial composition in family level of all the samples, (b) bacterial composition in family level of the five soil types.

Fig 4. The bacterial relative abundance of main biosynthesis function (a) and the Pearson correlation analysis between OM fractions and major microbial functional genes (b). The "**" meant significance was less than 0.01, and "*" meant significance was less than 0.05.in the five soil types.

Fig 5. The bacterial relative abundance of cytochrome c biosynthesis function in aerobic respiration based on the MetaCyc (https://metacyc.org/) metabolic pathway prediction.

Fig 6. The proportions of C fractions (a) and N fractions (b) in the five land-use types.

Araphic abstract

Click here to access/download;Figure;graphic abstract2.tif

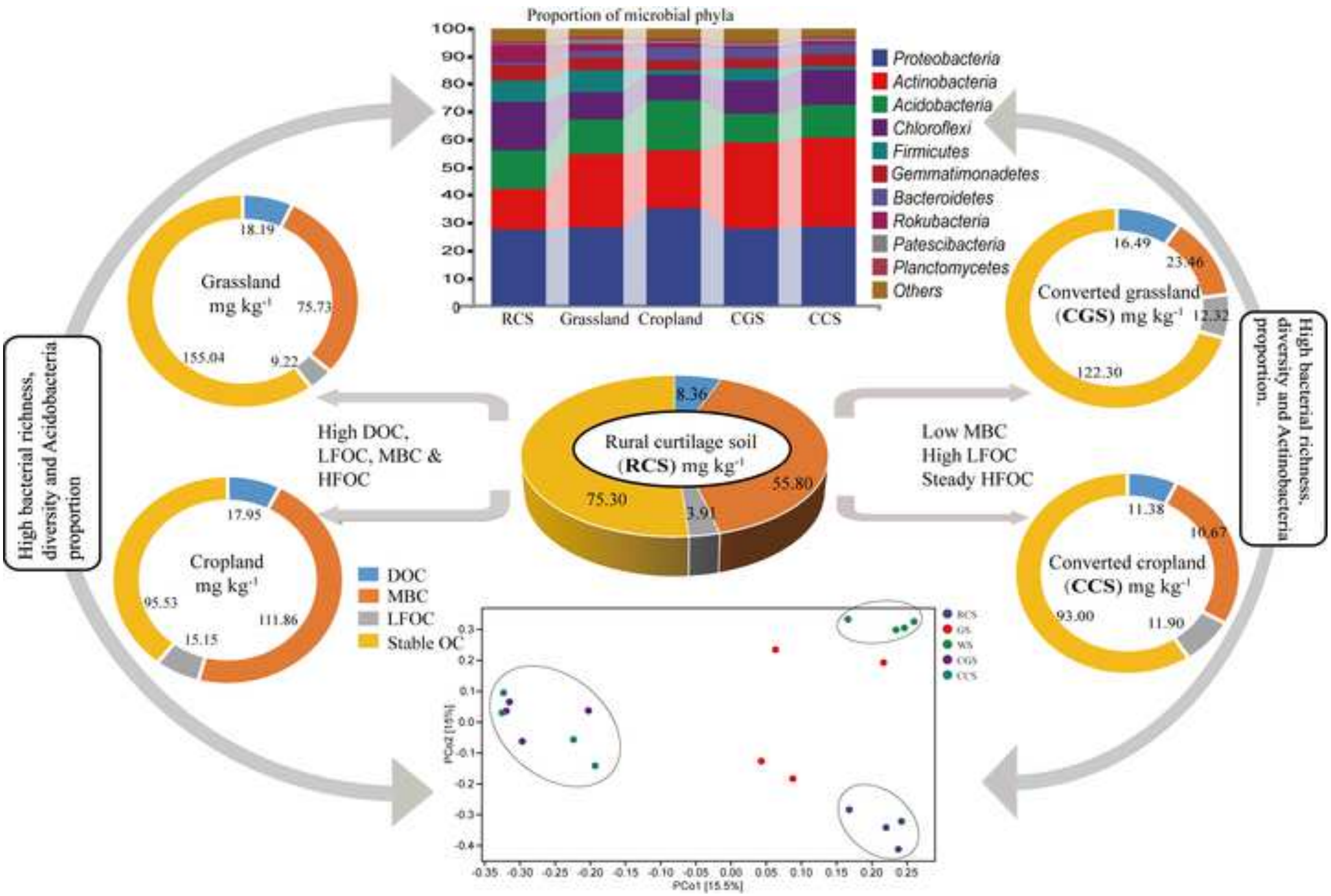

Fig 1

Click here to access/download;Figure;Fig 1.tif

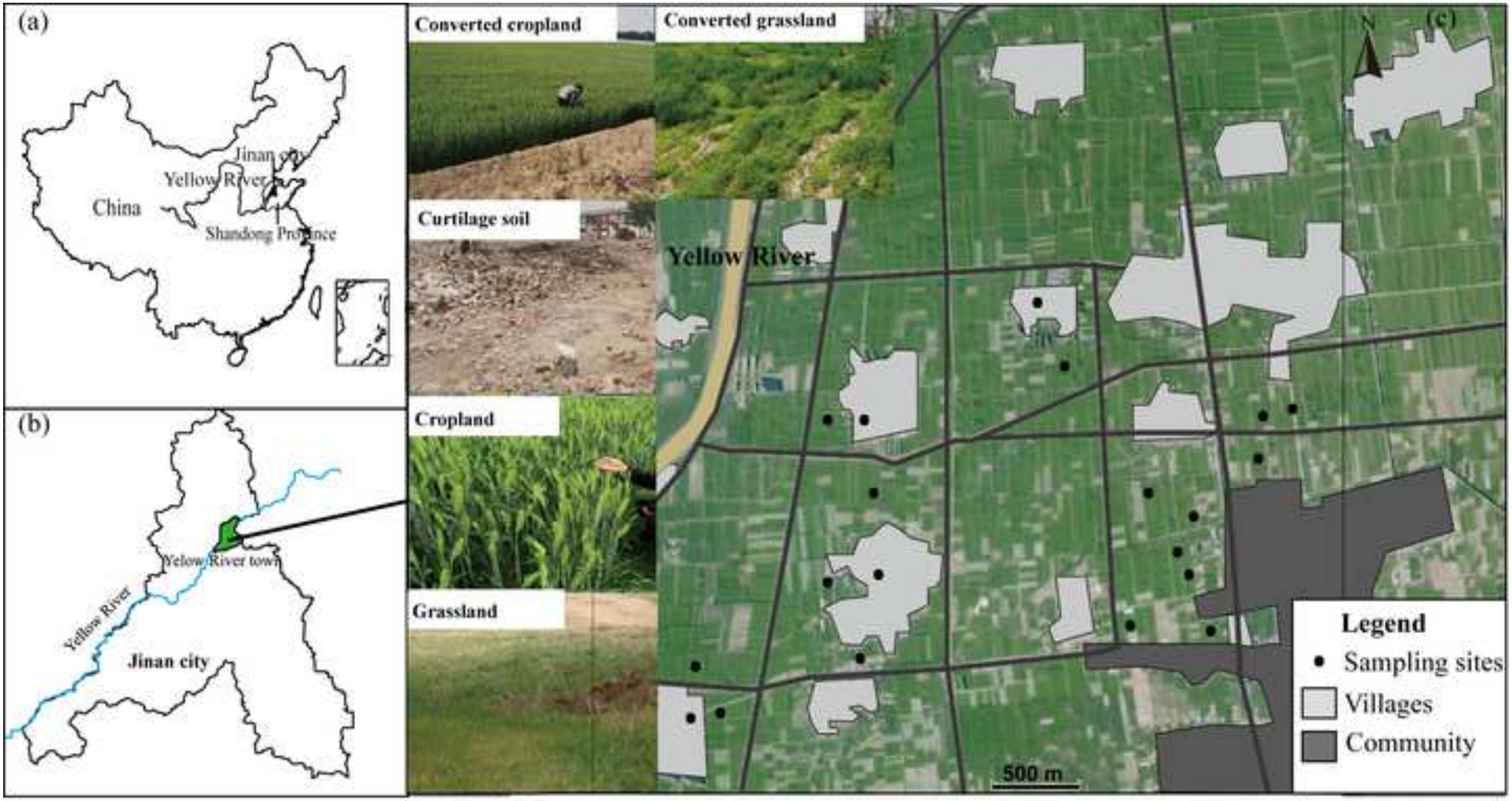

Fig 2

Click here to access/download;Figure;Fig 2.tif

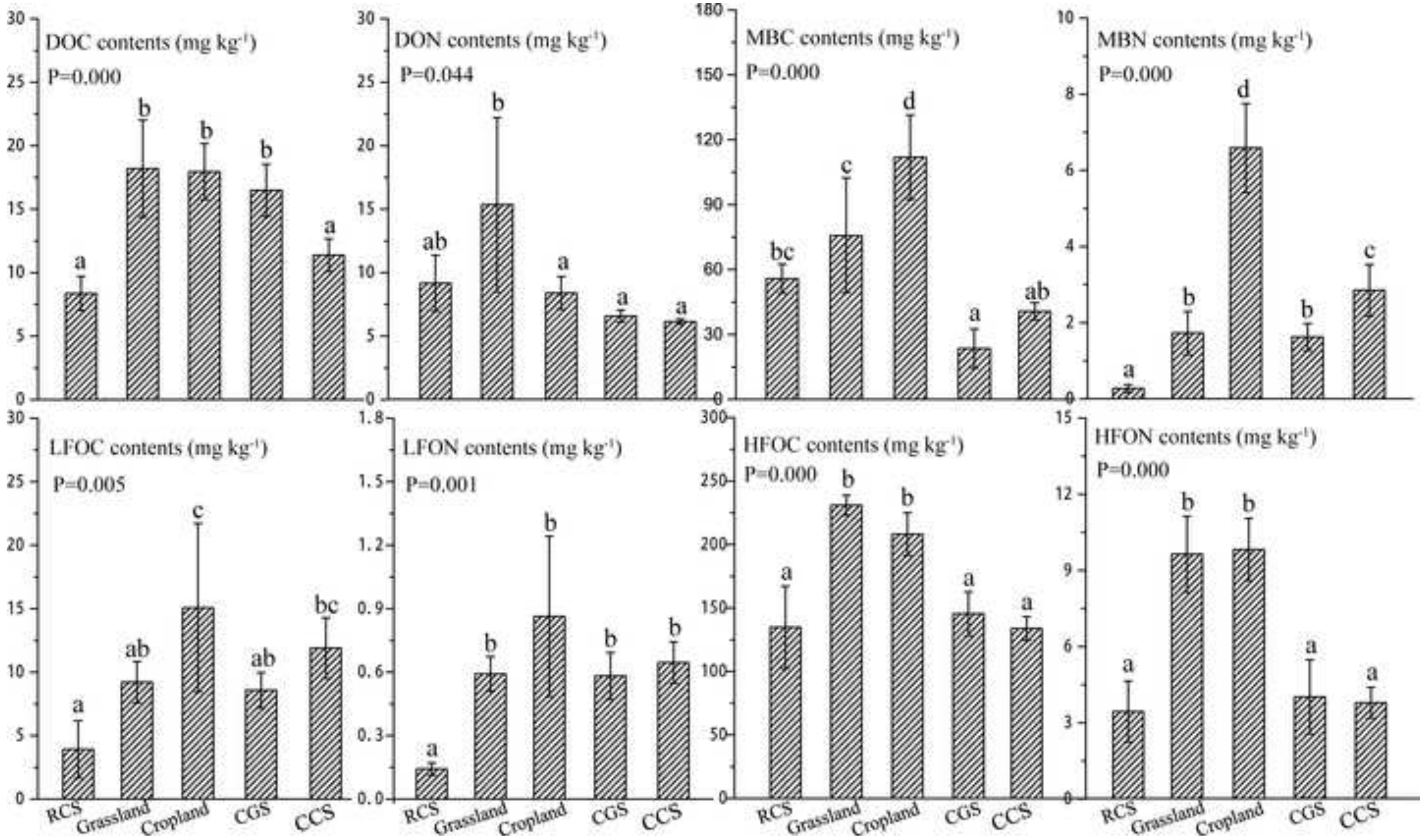

Fig 3

Click here to access/download;Figure;Fig 3.tif

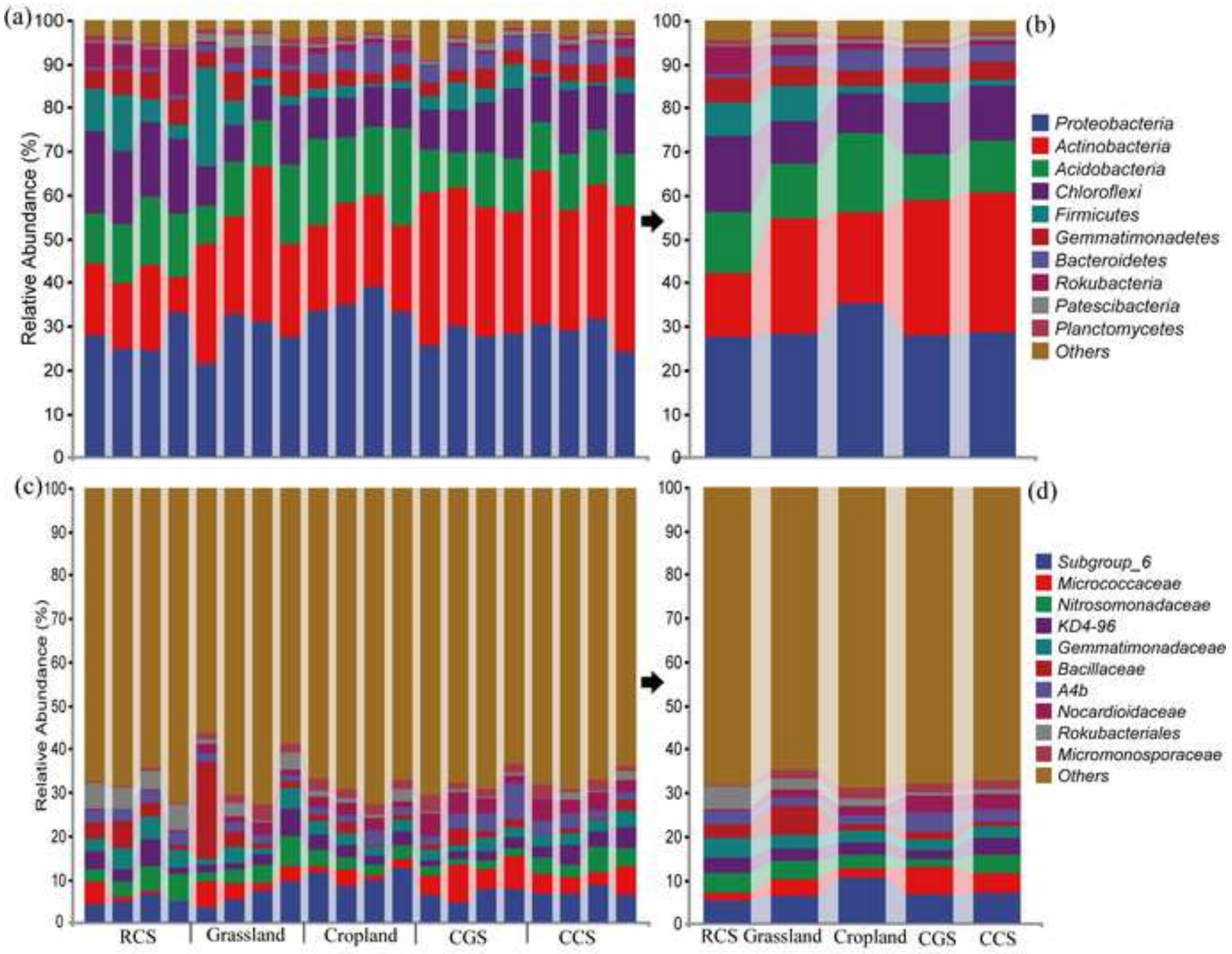

Click here to access/download;Figure;Fig. 4.tif

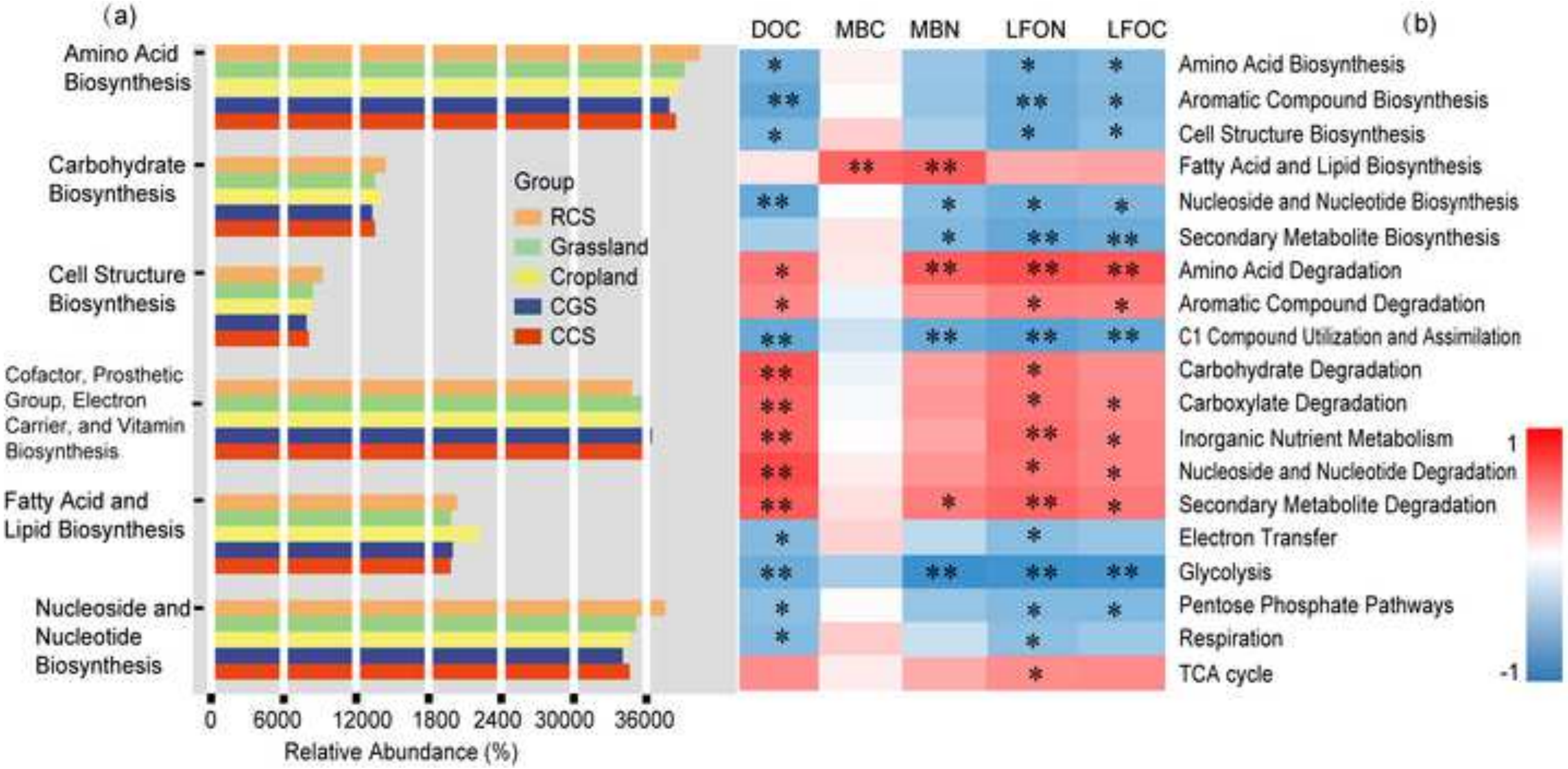

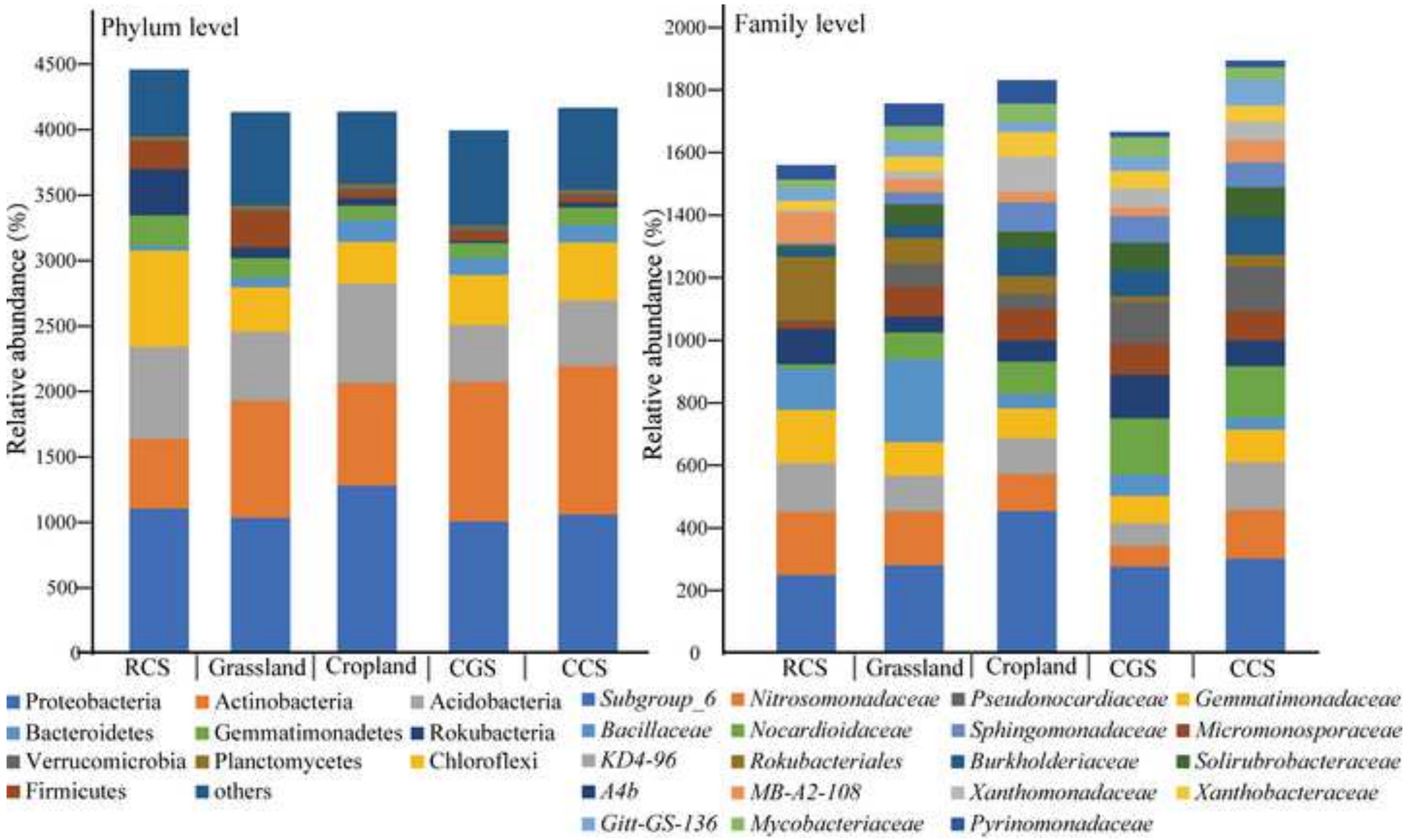

Phylum level
Relative abundance (%)
4500
4000
3500
3000
2500
2000
1500
1000
500
0
RCS
Grassland
Cropland
CGS
CCS
Proteobacteria
Actinobacteria
Acidobacteria
Bacteroidetes
Gemmatimonadetes
Rokubacteria
Verrucomicrobia
Planctomycetes
Chloroflexi
Firmicutes
others
Family level
Relative abundance (%)
2000
1800
1600
1400
1200
1000
800
600
400
200
0
RCS
Grassland
Cropland
CGS
CCS
Subgroup_6
Nitrosomonadaceae
Pseudonocardiaceae
Gemmatimonadaceae
Bacillaceae
Nocardioidaceae
Sphingomonadaceae
Micromonosporaceae
KD4-96
Rokubacteriales
Burkholderiaceae
Solirubrobacteraceae
A4b
MB-A2-108
Xanthomonadaceae
Xanthobacteraceae
Gitt-GS-136
Mycobacteriaceae
Pyrinomonadaceae

Fig 6

Click here to access/download;Figure;Fig. 6.tif

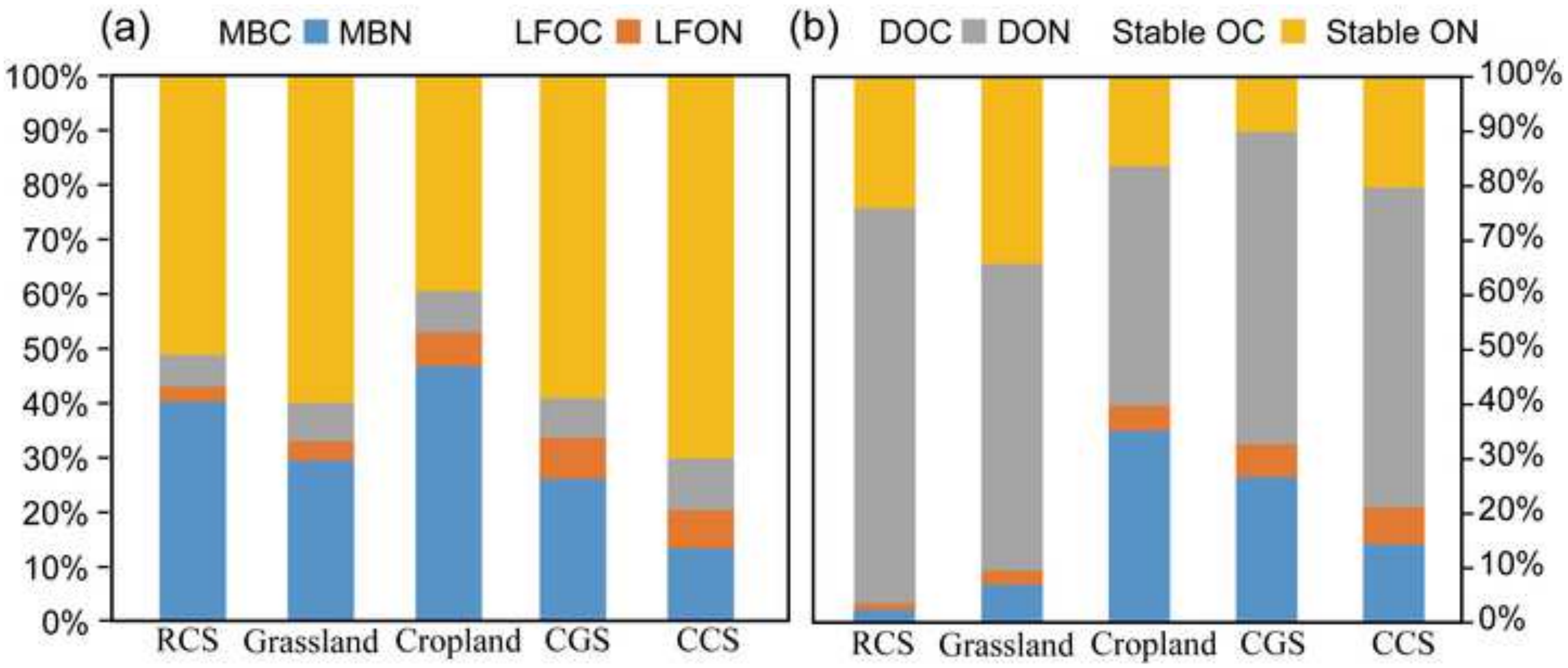

Click here to access/download
Supporting Information
Supplementary material.docx
