# Supplementary material for: Soil organic carbon fraction accumulation and bacterial characteristics in curtilage soil: Effects of land conversion and land use
Source: PLoS One. 2023 Apr 6;18(4):e0283802. doi: 10.1371/journal.pone.0283802 (PMC10079021; doi:10.1371/journal.pone.0283802)
Supplement: Attachment — Submitted filename: response to reviewers.docx [file pone.0283802.s009.docx]

**Response to the editor and reviewers’ suggestions point-by-point**

Responses to Editor's specific comments:

1. Please ensure that your manuscript meets PLOS ONE's style requirements, including those for file naming. The PLOS ONE style templates can be found at

https://journals.plos.org/plosone/s/file?id=wjVg/PLOSOne_formatting_sample_main_body.pdf and

https://journals.plos.org/plosone/s/file?id=ba62/PLOSOne_formatting_sample_title_authors_affiliations.pdf

We have revised and rechecked the manuscript format based on the files you given. Thanks very much!

2. We suggest you thoroughly copyedit your manuscript for language usage, spelling, and grammar. If you do not know anyone who can help you do this, you may wish to consider employing a professional scientific editing service.

Thanks for you suggestions, language usage of this manuscript is revised by Editage Company (www.editage.com) that you recommended. Thanks for your coupon.

3. We note that the grant information you provided in the ‘Funding Information’ and ‘Financial Disclosure’ sections do not match.

When you resubmit, please ensure that you provide the correct grant numbers for the awards you received for your study in the ‘Funding Information’ section.

Thanks for your comments. We removed the funding information in “acknowledgement” part.

4. Thank you for stating the following financial disclosure:

   "Yes. This study is supported by the National Key Research and Development Plan “Research on the spatial optimization and layout of rural and communities” (NO. 2019YFD1100801), recieved by Dongxu Cui. Prof Cui participates in the study design, data analysis and manuscript writing."

Please state what role the funders took in the study.  If the funders had no role, please state: "The funders had no role in study design, data collection and analysis, decision to publish, or preparation of the manuscript."

If this statement is not correct you must amend it as needed.

Please include this amended Role of Funder statement in your cover letter; we will change the online submission form on your behalf.

Thanks for your comments. The relevant research of this manuscript is supported by The National key research and development program “Research on the spatial optimization and layout of rural and communities” (NO. 2019YFD1100801). Prof. Dongxu Cui is the program director. The funders had no role in study design, data collection and analysis, decision to publish, or preparation of the manuscript.

5. Thank you for stating the following in the Acknowledgments Section of your manuscript:

    "We thank Shanghai Personal Biotechnology Co., Ltd. (Shanghai, China) for microbial Illumina Sequencing. We also thank Dr. Junyu Dong for the revision. This study was supported by the National Key Research and Development Plan “Research on the spatial optimization and layout of rural and communities” (NO.

2019YFD1100801)."

We note that you have provided funding information that is not currently declared in your Funding Statement. However, funding information should not appear in the Acknowledgments section or other areas of your manuscript. We will only publish funding information present in the Funding Statement section of the online submission form.

Please remove any funding-related text from the manuscript and let us know how you would like to update your Funding Statement. Currently, your Funding Statement reads as follows:

   "Yes. This study is supported by the National Key Research and Development Plan “Research on the spatial optimization and layout of rural and communities” (NO. 2019YFD1100801), recieved by Dongxu Cui. Prof Cui participates in the study design, data analysis and manuscript writing."

Please include your amended statements within your cover letter; we will change the online submission form on your behalf.

Thanks for your comment.We removed the funding information in “acknowledgement” part. This study is supported by the National Key Research and Development Plan “Research on the spatial optimization and layout of rural and communities” (NO. 2019YFD1100801). Prof. Dongxu Cui is the program director. The funders had no role in study design, data collection and analysis, decision to publish, or preparation of the manuscript.

6. Thank you for stating the following in your Competing Interests section:

    "NO authors have competing interests"

Please complete your Competing Interests on the online submission form to state any Competing Interests. If you have no competing interests, please state "The authors have declared that no competing interests exist.", as detailed online in our guide for authors at http://journals.plos.org/plosone/s/submit-now

 This information should be included in your cover letter; we will change the online submission form on your behalf.

Thanks for your comment. "The authors have declared that no competing interests exist." is stated in the revised cover letter.

7. We note that you have stated that you will provide repository information for your data at acceptance. Should your manuscript be accepted for publication, we will hold it until you provide the relevant accession numbers or DOIs necessary to access your data. If you wish to make changes to your Data Availability statement, please describe these changes in your cover letter and we will update your Data Availability statement to reflect the information you provide.

Thanks for your comment. We can update the microbial data to NCBI when submit the revised manuscript. This has stated in the revised cover letter.

8. PLOS requires an ORCID iD for the corresponding author in Editorial Manager on papers submitted after December 6th, 2016. Please ensure that you have an ORCID iD and that it is validated in Editorial Manager. To do this, go to ‘Update my Information’ (in the upper left-hand corner of the main menu), and click on the Fetch/Validate link next to the ORCID field. This will take you to the ORCID site and allow you to create a new iD or authenticate a pre-existing iD in Editorial Manager. Please see the following video for instructions on linking an ORCID iD to your Editorial Manager account: <https://www.youtube.com/watch?v=_xcclfuvtxQ>

Thanks for your comment, Prof. Dongxu Cui has registered the ORCID iD, which is 0000-0003-1962-1459.

9. We note that Figure 1 in your submission contain [map/satellite] images which may be copyrighted. All PLOS content is published under the Creative Commons Attribution License (CC BY 4.0), which means that the manuscript, images, and Supporting Information files will be freely available online, and any third party is permitted to access, download, copy, distribute, and use these materials in any way, even commercially, with proper attribution. For these reasons, we cannot publish previously copyrighted maps or satellite images created using proprietary data, such as Google software (Google Maps, Street View, and Earth). For more information, see our copyright guidelines: <http://journals.plos.org/plosone/s/licenses-and-copyright.>
10. We require you to either (a) present written permission from the copyright holder to publish these figures specifically under the CC BY 4.0 license, or (b) remove the figures from your submission:
11. a. You may seek permission from the original copyright holder of Figure 1 to publish the content specifically under the CC BY 4.0 license.
12. We recommend that you contact the original copyright holder with the Content Permission Form (http://journals.plos.org/plosone/s/file?id=7c09/content-permission-form.pdf) and the following text:
13. “I request permission for the open-access journal PLOS ONE to publish XXX under the Creative Commons Attribution License (CCAL) CC BY 4.0 (http://creativecommons.org/licenses/by/4.0/). Please be aware that this license allows unrestricted use and distribution, even commercially, by third parties. Please reply and provide explicit written permission to publish XXX under a CC BY license and complete the attached form.”
14. Please upload the completed Content Permission Form or other proof of granted permissions as an "Other" file with your submission.
15. In the figure caption of the copyrighted figure, please include the following text: “Reprinted from [ref] under a CC BY license, with permission from [name of publisher], original copyright [original copyright year].”
16. b. If you are unable to obtain permission from the original copyright holder to publish these figures under the CC BY 4.0 license or if the copyright holder’s requirements are incompatible with the CC BY 4.0 license, please either i) remove the figure or ii) supply a replacement figure that complies with the CC BY 4.0 license. Please check copyright information on all replacement figures and update the figure caption with source information. If applicable, please specify in the figure caption text when a figure is similar but not identical to the original image and is therefore for illustrative purposes only.
17. The following resources for replacing copyrighted map figures may be helpful:
18. USGS National Map Viewer (public domain): http://viewer.nationalmap.gov/viewer/
19. The Gateway to Astronaut Photography of Earth (public domain): http://eol.jsc.nasa.gov/sseop/clickmap/
20. Maps at the CIA (public domain): https://www.cia.gov/library/publications/the-world-factbook/index.html and https://www.cia.gov/library/publications/cia-maps-publications/index.html
21. NASA Earth Observatory (public domain): http://earthobservatory.nasa.gov/
22. Landsat: http://landsat.visibleearth.nasa.gov/
23. USGS EROS (Earth Resources Observatory and Science (EROS) Center) (public domain): http://eros.usgs.gov/#
24. Natural Earth (public domain): <http://www.naturalearthdata.com/>

Thanks for your suggestions. We drew the Figure 1 by referring the Geospatial Data Cloud site, Computer Network Information Center, Chinese Academy of Sciences. (<http://www.gscloud.cn>). In the revised manuscript, we revised the Figure 1 and noted the referring information.

25. Please include captions for your Supporting Information files at the end of your manuscript, and update any in-text citations to match accordingly. Please see our Supporting Information guidelines for more information: http://journals.plos.org/plosone/s/supporting-information.
26. [Note: HTML markup is below. Please do not edit.]

Thanks for your comment. Figure captions were inserted immediately after the first paragraph in which the figure is cited. We added the Supporting Information in the revised manuscript, and figure and table captions in supplementary file were listed.

**Response to the editor and** **reviewers’ suggestions point-by-point**

Responses to reviewers’ specific comments:

Reviewer #1: Dear Authors,

The manuscript require rigorous effort to improve its quality of presentation. Specifically, suggestions have been given in the reviewed manuscript itself.

Answer: We greatly appreciate the constructive and detailed comments. The English language of the whole manuscript was revised and checked by Editage Company and all the authors. All your suggestions were adopted and the manuscript was revised based on your suggestions. We hope that our revision and answers will enable our manuscript to win your satisfaction. And we would be happy to do whatever is required if further revision is necessary.

In general, the suggestions have been mentioned below:

1. Abstract: Rewrite abstract. Do not repeat the sentences, quantify your finding to understand how LUC has improved the OM fractions (1-2 lines). Also, first mention about results of SOC fraction followed by microbial high-throughput analysis. Use full and abbreviations appropriately.

Answer: Thanks for your comment. We thoroughly revised the “Abstract” part and reduced the usage of abbreviations. The revised Abstract was showed below.

**Abctract:**

Conversion of curtilage into cropland or grassland can have substantial effects on soil nutrition and microbial activities; however, these effects remain ambiguous. This is the first study to compare the soil organic carbon (SOC) fractions and bacterial communities in rural curtilage, converted cropland, and grassland compared with cropland and grassland. This study determined the light fraction (LF) and heavy fraction (HF) of organic carbon (OC), dissolved organic carbon (DOC), microbial biomass carbon (MBC), and the microbial community structure by conducting a high-throughput analysis. Curtilage soil had significantly lower OC content, the DOC, MBC, LFOC and HFOC of grassland and cropland soils were 104.11%, 55.58%, 264.17%, and 51.04% higher than curtilage soil averagely. Cropland showed notably high bacterial richness and diversity, with Proteobacteria (35.18%), Actinobacteria (31.48%), and Chloroflexi (17.39%) predominating in cropland soil, grassland soil, and curtilage soil, respectively. Moreover, DOC and LFOC contents of converted cropland and grassland soils were 47.17% and 148.65% higher than curtilage soil while MBC content was 46.24% lower than curtilage soil averagely. Land conversion showed more significant effects on microbial composition than land-use differences. The abundant Actinobacteria and Micrococcaceae population and the low MBC contents indicated a “hungry” microbial state in the converted soil, whereas the high MBC content, Acidobacteria proportion, and relative abundance of functional genes in the fatty acid and lipid biosynthesis indicated a “fat” microbial state in cropland. This study contributes to the improvement of soil fertility and the comprehension and efficient use of curtilage soil.

2. Introduce your topic accurately. Do not use irreverent information that have no scientific background. Use appropriate unit for presentation. Several statements have not been found linked to generated proper hypothesis. Go through attached reviewed manuscript for more clarification.

Answer: Thanks for your comment. For the Introduction part, the sentence “Rural revitalization strategy is firstly reported on October 18th, 2017, it aims to improve the natural, social and economic levels in rural areas” were deleted, the unit was revised. After careful revision, several sentences were revised and polished, the detailed changes were showed in the revised manuscript.

3. Methodology should be clear. Use full form of any words along with its abbreviation at first time. Write about indices and its equation. How it was determined? No such information is present in the M&M. Use appropriate unit. Explain about new terminology. such as curtilage soil, tidal soil.

Answer: Thanks for you comment. We explained the curtilage soil, converted cropland soil and converted grassland soil in the revised manuscript. Meanwhile, we added the full name along with its abbreviation. In addition, we revised the description of experimental procedures and checked the English writing. The detailed changes were showed in the revised manuscript.

4. Result and discussion: Not written well. Many information present in R&D have not been mentioned in M&M.

Answer: Thanks for you comments. We revised this part by polishing the language, replenishing the analysis methods in M&M, improving the writing logic, etc. The detailed changes were showed in the text.

5. Improve the resolution of figures.

Answer: Thanks for your comments, we improved the resolution of figures.

6. Conclusion: Rewrite it and concise the conclusion.

Answer: Thanks for your suggestion. We simplified the Conclusions in the revised manuscript, which showed below.

We investigated the relationships between OM fractions and microbial communities, C and N fractions, microbial composition, and functional gene abundance across five land-use types. Curtilage soil (CS) had the lowest OM fractions but higher LFOM and microbial-OM levels, whereas cropland and grassland soil had comparable HFOM and DOC contents. Cropland exhibited a high bacterial richness and diversity, with Proteobacteria, Actinobacteria, and Chloroflexi predominating in cropland, grassland, and CS, respectively. Cropland and grassland converted from curtilage had higher LFOM and MBN content, the same level of HFOM and DOM, and a significantly lower MBC than CS. Different land types suggested various microbial states based on the bacterial abundance and MBC contents. Therefore, the above results contributed to the poor growth of crops or grass on converted soil but vigorous wheat growth on cropland. This is the first study to describe SOM and bacterial community composition characteristics in rural curtilage soil and potential changes when converted to cropland and grassland. It improved the understanding and efficient use of curtilage converted land. Several measures should be conducted to activate and fatten the bacteria in curtilage soil.

Reviewer #2: 1. Abstract should be more focused with a line of research gap, hypothesis and few best quantitative data.

Answer: Thanks for your suggestions, we modified the text in Abstract and improved its writing logic. Moreover, we added the relevant data.

2. Very recent relevant citation must be incorporate in introduction and discussion section.

Answer: Thanks for your suggestions, we added several recent published papers in the revised manuscript.

3. Line no. 137: Rewrite the “℃”,

Answer: Thanks for your suggestions, we revised it into “°C”.

4. Line no. 173: Only write 10g instead of “10.00 g”

Answer: Thanks for your suggestions, we revised it as you suggested.

5. Materials and methods: Write a bit details of DNA sequencing steps you have followed like trimming, no of reads generated, contig, aligning etc.

Answer: Thanks for the suggestions, we added the relevant description in the revised manuscript, which also listed below.

The paired-end reads were excised by Qiime2 cutadapt trim-paired process, and the sequence of the unmatched primer was discarded. The paired-end reads were merged to obtain the raw tags. Qiime quality control process was used to filter the raw tags and then the high-quality sequence was obtained. Chimeric sequences were detected and removed by using Qiime dada2 denoise-paired process. Then, UPARSE (www.drive5.com/uparse/) was used to cluster the high-quality sequences into operational taxonomic units with 97% similarity threshold. Finally, the operational taxonomic units were taxonomically annotated against the SILVA132 reference database (http://www.arb-silva.de/).

6. Result and discussion should follow as per materials and methods (M&M) sequence. Therefore, resequence your M&M like-wise; otherwise start discussion with the result of LFOM and HFOM.

Answer: Thanks for your suggestion. As you suggested, we moved the analysis of DOC and MBC ahead in M&M part.

7. Line no. 264-265: Reason for italicized the family name “family level, Subgroup 6, Micrococcaceae, Nitrosomonadaceae, KD4-96, and Gemmatimonadaceae”??

Answer: Thanks for your question, it’s unnecessary to italicized the family name, thus we revised them in the whole manuscript.

8. Elaborately discuss the section “Bacterial composition in different soil types” with recent reports and try to establish a strong correlation with perspective treatments.

Answer: Thanks for your suggestion. We added the relevant papers and discussed the effects of soil-type differences in the revised manuscript.

9. In Fig 1: Direction map should layer in this figure.

Answer: Thanks for your suggestion. We revised the Figure 1 in the revised version.

10. Conclusion should be modified as per the best result and a line of recommendation.

Answer: Thanks for your suggestion. We simplified the Conclusions in the revised manuscript, which showed below.

We investigated the relationships between OM fractions and microbial communities, C and N fractions, microbial composition, and functional gene abundance across five land-use types. Curtilage soil (CS) had the lowest OM fractions but higher LFOM and microbial-OM levels, whereas cropland and grassland soil had comparable HFOM and DOC contents. Cropland exhibited a high bacterial richness and diversity, with Proteobacteria, Actinobacteria, and Chloroflexi predominating in cropland, grassland, and CS, respectively. Cropland and grassland converted from curtilage had higher LFOM and MBN content, the same level of HFOM and DOM, and a significantly lower MBC than CS. Different land types suggested various microbial states based on the bacterial abundance and MBC contents. Therefore, the above results contributed to the poor growth of crops or grass on converted soil but vigorous wheat growth on cropland. This is the first study to describe SOM and bacterial community composition characteristics in rural curtilage soil and potential changes when converted to cropland and grassland. It improved the understanding and efficient use of curtilage converted land. Several measures should be conducted to activate and fatten the bacteria in curtilage soil.

11. Spacing issue should be carefully checked, besides, please revise whole MS once again for at least fluent scientific English language.

Answer: We greatly appreciate the constructive and detailed comments. The English language of the whole manuscript was revised and checked by Editage Company and all the authors. All your suggestions were adopted and the manuscript was revised based on your suggestions. We hope that our revision and answers will enable our manuscript to win your satisfaction. And we would be happy to do whatever is required if further revision is necessary.
