# Supplementary material for: Soil organic carbon fraction accumulation and bacterial characteristics in curtilage soil: Effects of land conversion and land use
Source: PLoS One. 2023 Apr 6;18(4):e0283802. doi: 10.1371/journal.pone.0283802 (PMC10079021; doi:10.1371/journal.pone.0283802)
Supplement: Attachment — Submitted filename: COMMENTS OF THE REFREE.docx [file pone.0283802.s010.docx]

**COMMENTS OF THE REFREE (PONE-D-22-15913_R1)**

Dear Authors,

The manuscript still requires some of your quality effort to improve its quality of presentation.

The study is good and is of general importance. The introduction and background are not well structured. The experimental data and material is adequate but representation of the data should be more crispy. Statistical analysis of data is perfect and very sound. Result and discussion part should be rewritten and more focused and elaborative with giving explanation like why the abovesaid phyla are more dominant under specific condition, etc. Specifically, suggestions have been given as following:

P1. Introduce your topic accurately. This part should be crisper indicating the research gap and why the study is so much important on global perspective and need to be more focused.

P2. Methodology should be clear. Use full form of any words along with its abbreviation at first time (DOC and DON).

P3. Write about indices and its equation. How it was determined? No such information is present in the M&M. Use appropriate unit with reference.

P4. In line no. 110. (<http://www.gscloud.cn>): try to specify the area or it should be readily available and easy to locate.

P5. In line no. 135. Write full form of LFOC and HFOC. Mention the standard procedure of their calculation with proper references. How they are separated by using 0.043 mm sieve and give proper reference for that.

P6. How LFOC and HFOC are related with LFOM and HFOM: Mention it. Which fraction is actually measured and how? Give detailed procedure. If only LFOC and HFOC are measured then how it is converted to LFOM and HFOM as they are not synonymous. Similarly for DOM and DOC?

P7. In line no. 209. Table1. The value should be upto 4 digit.

P8. In line no.278: in place of connection, “relationship” may be written.

P9. Result and discussion: Not written well. Many information present in R&D have not been mentioned in M&M

P10. Improve the resolution of figures.

P11. The discussion part was written very superficially and it should be very focused and elaborative and if, it was recorded or found, then try to give a proper explanation that why it was happened.

P12. Conclusion: Conclusion should be modified as per the best result and a line of recommendation. The conclusion part should not be summary of the result of the research work. You should include how the information generated by you would be useful at global perspective.

P13. In line no.374-75: “therefore, …….cropland”: what does it mean, please clarify?

P14. In figure 6: what do you mean by stable OC and ON. Mention its detail in the text and material and methods part as well.

Overall, this study in this stage is not suitable for publication unless authors provide the details mentioned above.
