# Supplementary material for: Soil organic carbon fraction accumulation and bacterial characteristics in curtilage soil: Effects of land conversion and land use
Source: PLoS One. 2023 Apr 6;18(4):e0283802. doi: 10.1371/journal.pone.0283802 (PMC10079021; doi:10.1371/journal.pone.0283802)
Supplement: Attachment — Submitted filename: Response to Reviewers-R2.doc [file pone.0283802.s011.doc]

**Response to Reviewers**

Reviewer #3:

Dear Authors,

The manuscript still requires some of your quality effort to improve its quality of presentation.

The study is good and is of general importance. The introduction and background are not well structured. The experimental data and material is adequate but representation of the data should be more crispy. Statistical analysis of data is perfect and very sound. Result and discussion part should be rewritten and more focused and elaborative with giving explanation like why the abovesaid phyla are more dominant under specific condition, etc. Specifically, suggestions have been given as following:

Overall, this study in this stage is not suitable for publication unless authors provide the details mentioned above.

Answer: We greatly appreciate your encouragement and constructive suggestions. We enriched the research background and importance in the Introduction part, revised and improved the representation and wording in the Materials and Methods part, replenished the relevant references of index calculation. In Result and Discussion part, we discussed the relationships of dominant bacteria and land types. We hope that our revision and answers will enable our manuscript to win your satisfaction.

P1. Introduce your topic accurately. This part should be crisper indicating the research gap and why the study is so much important on global perspective and need to be more focused.

Answer: Thanks for your suggestions. We restated the research background in China and the research importance in global scale. Meanwhile, we added relevant references to support the views. They were showed in the revised manuscript.

P2. Methodology should be clear. Use full form of any words along with its abbreviation at first time (DOC and DON).

Answer: Thanks for your suggestions. We revised them in the new revised manuscript.

P3. Write about indices and its equation. How it was determined? No such information is present in the M&M. Use appropriate unit with reference.

Answer: Thanks for your suggestions. We added the accordance of index calculation. The added part was: “Chao1 Index and Observed Species Index were calculated to determine bacterial richness, and Shannon Index was proceeded to analyze the bacterial diversity (http://scikit-bio.org/docs/latest/generated/skbio.diversity.alpha.html#module-skbio.diversity.alpha) [28-30].”

P4. In line no. 110. ([http://www.gscloud.cn](http://www.gscloud.cn/)): try to specify the area or it should be readily available and easy to locate.

Answer: Thanks for your suggestions. We added the detailed data identification, which was showed: “ The data information is obtained from Landset 8 OLI_TIRS with data identification of LC81220342021147LGN00, which is provided by Geospatial Data Cloud site, Computer Network Information Center, Chinese Academy of Sciences. ([http://www.gscloud.cn](http://www.gscloud.cn/)).”

P5. In line no. 135. Write full form of LFOC and HFOC. Mention the standard procedure of their calculation with proper references. How they are separated by using 0.043 mm sieve and give proper reference for that.

Answer: Thanks for your suggestions. We added the full name of LFOC and HFOC. Meanwhile, we added detailed analyzing steps of the soil stratification, OM fraction separation and fraction determination. Detailed were showed: “Samples were air-dried, ground, and sieved through a 0.9 mm sieve. Subsequently, 10 g of each soil sample was weighed and combined with 40 mL of a 1.85 g·mL^-1^ sodium iodide solution for the soil stratification [24]. The light fraction (LF) was afloat and the heavy fraction (HF) was deposited [25]. The 10-min of vltrasonic oscillation and 10-min centrifugation were conducted with the mixtures for absolutely stratification. Then the LF was filtered through 0.043-mm brass sieve. The procedure was repeated 3 times to ensure complete separation. Then, by adding the 0.1 M the calcium chloride solution instead of sodium iodide solution, the procedure was repeated 5-6 times until all I- reactions ceased. The diluted hydrochloric acid (1:10 of volume) was added to remove soil inorganic carbon and repeated the above step. Moreover, this step was repeated once or twice by adding ultrapure water until all Cl- reactions ceased. Finally, the LF and HF were transferred into weighed beakers and oven-dried at 40 °C. LF and HF were weighed as LFOM and HFOM respectively. Both the elements of C and N in LFOM and HFOM were determined by using an element analyzer (Vario EL III, Elementar Analysensysteme, Germany), and the contents of LFOC, LFON, HFOC and HFON were calculated [10].”

P6. How LFOC and HFOC are related with LFOM and HFOM: Mention it. Which fraction is actually measured and how? Give detailed procedure. If only LFOC and HFOC are measured then how it is converted to LFOM and HFOM as they are not synonymous. Similarly for DOM and DOC?

Answer: Thanks for your suggestions. We supplied the relations between LFOC, HFOC and LFOM, HFOM, rewrote the detailed procedure. Meanwhile, we also added the detailed analyzing steps of DOC and DON, and explained the relations between DOC, DON and DOM. This part was revised as follows:

**Analysis of DOC and MBC**

The chloroform fumigation method was conducted on all the soil samples to kill the microbes [21]. A vacuum pump was used to boil 20 mL of chloroform in the drying cabinet after adding 10 g of fresh soil and the sodium hydrate. After vacuuming out the gasified chloroform, the drying cabinet was left overnight to eliminate any lingering microorganisms. Meanwhile, a weighed portion of fresh soil and sodium hydrate solution was placed inside a drying cabinet as control. Both the fumigated and control soils were mixed with 2-M potassium chloride (KCl) solution at 1:5 ratio. After oscillation for 1 h at speed of 200 r/min, the mixtures were centrifugation at 4500 r/min for 10 min and filtration with filter membrane of 0.045 μm. The dissolvable organic matter in supernatant of the control soil was identified as soil DOM, and that of the fumigated soil was the sum of microbial biomass organic matter and soil dissolved organic matter. The C and N contents of the supernatant were determined using a C/N analyzer (ANALYTIKJENA MULTI N/C 3100, Elementar Analysensysteme, Germany), and C and N contents in supernatant of the control soil were the DOC and DON respectively. The MBC and MBN contents were the remainders of C and N in fumigated soil subtracted with those in controlled soil respectively.

In addition, 10 g of fresh soil was extracted using a 2-M of KCl solution in a 1:5 ratio. The organic carbon and organic nitrogen contents of the leaching solution were analyzed, and corresponding DOC and DON contents were calculated [15]. Moreover, soil pH was determined by a pH meter.

**LFOM and HFOM analysis**

Samples were air-dried, ground, and sieved through a 0.9 mm sieve. Subsequently, 10 g of each soil sample was weighed and combined with 40 mL of a 1.85 g·mL^-1^ sodium iodide solution for the soil stratification [24]. The light fraction (LF) was afloat and the heavy fraction (HF) was deposited [25]. The 10-min of vltrasonic oscillation and 10-min centrifugation were conducted with the mixtures for absolutely stratification. Then the LF was filtered through 0.043-mm brass sieve. The procedure was repeated 3 times to ensure complete separation. Then, by adding the 0.1 M the calcium chloride solution instead of sodium iodide solution, the procedure was repeated 5-6 times until all I^-^ reactions ceased. The diluted hydrochloric acid (1:10 of volume) was added to remove soil inorganic carbon and repeated the above step. Moreover, this step was repeated once or twice by adding ultrapure water until all Cl^-^ reactions ceased. Finally, the LF and HF were transferred into weighed beakers and oven-dried at 40 °C. LF and HF were weighed as LFOM and HFOM respectively. Both the elements of C and N in LFOM and HFOM were determined by using an element analyzer (Vario EL III, Elementar Analysensysteme, Germany), and the contents of LFOC, LFON, HFOC and HFON were calculated [10].

P7. In line no. 209. Table1. The value should be upto 4 digit.

Answer: Thanks for your comments. We revised the Table 1 as you suggested.

P8. In line no.278: in place of connection, “relationship” may be written.

Answer: Thanks for your comments. We revised the Table 1 as you suggested.

P9. Result and discussion: Not written well. Many information present in R&D have not been mentioned in M&M

Answer: Thanks for your comments. We rechecked the correspondence between results of R&D and analyzing steps of M&M, and supplied the relevant procedure. The details were showed in the revised manuscript.

P10. Improve the resolution of figures.

Answer: Thanks for your suggestions. We rechecked and improved all the figure resolution.

P11. The discussion part was written very superficially and it should be very focused and elaborative and if, it was recorded or found, then try to give a proper explanation that why it was happened.

Answer: Thanks for your suggestions. We added the relevant discussion with several references of previous studies. We supplied relevant proper explanations and our deduction. The detailed were showed in the revised manuscript.

P12. Conclusion: Conclusion should be modified as per the best result and a line of recommendation. The conclusion part should not be summary of the result of the research work. You should include how the information generated by you would be useful at global perspective.

Answer: Thanks for your suggestions.we rewrote the Conclusion part as you suggested. The detailed was showed below.

**Conclusions**

With the process of rural relocation and combination, the soil quality of curtilage-converted land should be focused. This is the first study to describe SOM and bacterial community composition characteristics in rural curtilage soil and potential changes when converted to cropland and grassland. We find that the given bacterial composition promotes the degradation of microbial-derived C, resulting in a microbial “hungry” state and notably low MBC content in curtilage-converted grassland and cropland. Reversely, microorganism is in a “fat” state based on the significant abundance of biosynthesis related genes, high values of MBC content, bacterial richness and diversity. Therefore, effectively improving the soil MBC contents is the key to optimize soil quality. This study improves the understanding and efficient use of curtilage converted land. Several detailed measures should be conducted to activate and fatten the bacteria in curtilage soil.

P13. In line no.374-75: “therefore, …….cropland”: what does it mean, please clarify?

Answer: Thanks for your comment. We deleted this sentence and rewrote the Conclusion part.

P14. In figure 6: what do you mean by stable OC and ON. Mention its detail in the text and material and methods part as well.

Answer: Thanks for your comments. We are very sorry for the mistakes. We re-calculated the proportions of C and N fraction and redrew the Fig 6. based on the OC classification and C fraction separation steps, we determined the soil organic carbon (SOC) by summing the contents of DOC, LFOC and HFOC up, so was the soil organic nitrogen (SON). It is worth mentioning that proportion of MBC and MBN were the MBC: SOC ratio and MBN: SON ratio respectively. The detailed revision was showed below:

**Carbon accumulation potential**

The soil organic carbon (SOC) was the sum of DOC, LFOC and HFOC contents based on its separation steps, thus the proportion of each C fraction was determined by comparing it with the SOC, so was the proportion of each N fraction (Fig 6). For land-use types, grassland had the highest SOC content (258.39 mg·kg^-1^) than cropland and CS, among which, MBC proportion of grassland was significantly low. It may indicate the low efficiency of microbial metabolism, which facilitated the physicochemical wrapping and protection of OC particles, resulting in the OC accumulation and storage (Fig 6a and 6b). Ding et al. [56] also reported the advantage of microbial residue C accumulation in grassland. While CS showed the low proportions of DOC and LFOC, as well as the lowest contents of SOC. It illustrated small input limited the OC accumulation. For land conversion, CGS and CCS showed notably higher proportions of DOC and LFOC while lower MBC than CS, which led to markedly higher SOC accumulation in the converted soils. The findings also showed that low proportion of MBC was prone to C storage. Rafeza et al. [57] reported low emission of carbon dioxide with relatively low contents of MBC, which also confirmed the negative effect of MBC on OC accumulation. In contrast to grassland and cropland, CGS and CCS showed high LFOC, similar DOC while low MBC proportion. It suggested relatively low metabolism efficiency of LFOC in the converted soils.

The proportions of N fractions were also calculated and analyzed (Fig 6c and 6d). In this study, DON and HFON were the dominant fractions with proportion ranged of 43.87~72.37% and 36.48~51.52% respectively. For land types, soil organic nitrogen (SON) content was sorted as: grassland (25.57 mg·kg^-1^) > cropland (19.08 mg·kg^-1^) > CS (12.74 mg·kg^-1^) notably. Among that, CS showed significantly higher DON and lower HFON proportions, indicating the low stability. The cropland had extremely higher proportion of MBN (35.07%), which may benefit from increased microbial biomass, enzymatic activities, and ON utilization and consumption [58].

The C: N ratio is a good indicator of C and N mineralization in soil [59]. The notably high values of C: N in CGS (14.86) and CCS (15.34) in comparison to the low value of grassland (10.10) indicated a high rate of C and N mineralization. Theoretically, MBC: LFOC and MBC: DOC values could represent the quantity of unit MBC that degraded the total LFOC or produced the total DOC, respectively (S5 Fig). both the two values had significant differences among the five land-use types, which were also inter-related by an exponential function with an R^2^ of 0.736. Further analysis suggested that both LFOC consumption and DOC production were negatively regulated by Actinobacteria.

**Fig 6.** Proportions of C fractions and N fractions. The (a) showed proportions of DOC, LFOC, HFOC; (b) showed proportions of MBC and other organic carbon; (c) showed proportions of DON, LFON, HFON; (d) showed proportions of MBN and other organic nitrogen.
